# Supplementary material for: Excess winter mortality in Finland, 1971–2019: a register-based study on long-term trends and effect modification by sociodemographic characteristics and pre-existing health conditions
Source: BMJ Open. 2024 Feb 2;14(2):e079471. doi: 10.1136/bmjopen-2023-079471 (PMC10840061; doi:10.1136/bmjopen-2023-079471)
Supplement: Supplementary data [file bmjopen-2023-079471supp001.pdf]

SUPPLEMENTAL MATERIAL

Excess winter mortality in Finland in 1971-2019 – a register-based study on long-term trends and effect modification by sociodemographic characteristics and pre-existing health conditions

Ulla Suulamo, Hanna Remes, Lasse Tarkiainen, Mike Murphy, and Pekka Martikainen

Table S1. Categories of pre-existing health conditions.

Condition	Health care diagnosis, ICD-10 (Care register for health care HILMO, THL)	Refund categories, Finnish disease, or medicine codes (Register of Reimbursements for medicine purchases, Kela)
Dementia	F00-F03, F05.1, G30	307
Mental health disorders (excl. alcohol related)	F04-F99, excl. F10	Severe psychosis and other severe mental disorders (112)<br>Behavioural disorders in people with intellectual disabilities (113)
Diseases of the nervous system	G00-G29, G31-G99, excl. G31.2, G40.51, G62.1, G72.1	Parkinson's disease (110)<br>Epilepsy (111)<br>Multiple sclerosis (109)<br>Myasthenia gravis (108)<br>Neuralgia trigemini, neuralgia glossopharyngeal (119)<br>Narcolepsy (214)<br>Dimethyl fumarate, diroximel fumarate etc (157)<br>Retigabine and vigabatrin (181)<br>Brivaracetam, eslikarbazepin etc (182)<br>Lamotrigin and topiramate (183)<br>Rasagiline (193)<br>Rufinamide (199)
Diabetes	E10-E14	Diabetes (103)<br>Diabetes other than insulin therapy (215)
Cancer	C00-C97	Prostate cancer (116)<br>Breast cancer (115)
Diseases of the circulatory system (excl. alcohol related)	I00-I99, excl. I42.6	Chronic cardiac insufficiency (201)<br>Chronic coronary heart disease (206)<br>Chronic hypertension (205)<br>Chronic cardiac arrhythmias (207)
Diseases of the respiratory system	J00-J99, excl. J65	Chronic asthma and COPD (203)
Alcohol related causes	F10, G31.2, G40.51, G62.1, G72.1, I42.6, K29.2, K70, K86.0, K85.2, O35.4, X45	
Accidents and violence (excl. alcohol related)	V01-Y89, excl. X45	

Suulamo U, *et al. BMJ Open* 2024; 14:e079471. doi: 10.1136/bmjopen-2023-079471

Table S2. Changes in sociodemographic characteristics and pre-existing health conditions between 2000 and 2018, by age category, population aged 60 and over.

	2000/01 (%)				2018/19 (%)			
	**60–69**	**70–79**	**80–89**	**90–**	**60–69**	**70–79**	**80–89**	**90–**
**Sociodemographic characteristics**								
Mean age (sd)	64.3 (2.9)	74.1 (2.8)	83.6 (2.8)	92.2 (2.3)	64.5 (2.9)	73.8 (2.9)	83.8 (2.8)	92.5 (2.5)
Sex								
Men	47.0	39.0	28.1	20.9	48.5	45.6	37.6	24.9
Women	53.0	61.0	71.9	79.1	51.5	54.4	62.4	75.1
Income quartile								
1 (lowest)	25.0	25.0	25.0	25.2	25.0	25.0	25.0	24.8
2	25.0	25.0	25.0	24.9	25.0	25.0	25.0	25.0
3	25.0	25.0	25.0	24.9	25.0	25.0	25.0	25.1
4 (highest)	25.0	25.0	25.0	24.9	25.0	25.0	25.0	25.0
Living arrangement								
Household of >1	74.6	60.7	39.3	25.5	70.2	65.3	46.4	23.5
Alone	24.5	37.0	51.2	47.0	28.2	32.8	47.2	57.5
Institution	1.0	2.3	9.5	27.4	1.5	1.9	6.4	19.1
Large regions								
Coast	37.1	35.0	36.8	40.8	38.9	39.6	36.6	36.8
South	28.0	29.7	30.4	29.7	27.0	28.1	28.9	30.6
Center	19.0	19.6	18.9	17.7	17.8	17.6	18.9	18.2
North	16.0	15.7	13.9	11.8	16.3	14.7	15.7	14.4
**Pre-existing health conditions**								
Dementia	0.2	1.5	4.4	7.2	0.4	2.5	12.0	22.4
Mental health disorders	3.7	3.8	3.9	2.9	4.0	3.6	3.8	3.5
Cancer	3.3	5.1	5.7	4.4	5.1	8.8	11.0	9.3
Diseases of the nervous system	3.9	4.9	5.2	4.1	6.3	7.2	7.3	4.9
Diabetes	5.8	8.7	9.7	7.7	12.4	17.4	18.1	13.7
Circulatory	35.3	49.7	58.6	57.8	24.2	39.9	54.5	58.6
Respiratory	8.2	10.4	10.9	10.3	9.4	12.0	14.9	15.7
Alcohol related causes	0.4	0.2	0.1	0.1	0.7	0.4	0.2	0.0
Accidents and violence	3.3	4.4	7.2	10.2	5.0	6.2	9.6	13.7
Other than specified above	23.0	30.0	36.7	34.7	27.1	36.1	44.2	43.2
Any health condition	57.5	70.8	79.1	78.5	57.0	71.7	83.9	87.0
Total	100.0	100.0	100.0	100.0	100.0	100.0	100.0	100.0
N	488,751	372,305	152,561	22,026	729,831	530,492	248,299	50,543

Table S3. Relative risks of excess winter mortality by individual characteristics (95% CI) by age category. Finland 2000-2019, men and women aged 60 and over.

	**60–69** n=135,380	**70–79** n=224,573	**80–89** n=325,160	**90 and over** n=139,811
Winter-nonwinter rate ratio	1.06 (1.05, 1.07)	1.10 (1.09, 1.11)	1.14 (1.13, 1.15)	1.19 (1.17, 1.20)
**Sociodemographic characteristics**				
Sex				
Men	1.00	1.00	1.00	1.00
Women	1.01 (0.99, 1.03)	1.01 (0.99, 1.03)	**1.02 (1.00, 1.03)**	0.99 (0.97, 1.02)
Income quartile				
1 (lowest)	1.00	1.00	1.00	1.00
2	0.99 (0.96, 1.02)	1.00 (0.98, 1.03)	1.00 (0.98, 1.02)	1.01 (0.98, 1.04)
3	**0.96 (0.93, 0.99)**	1.01 (0.98, 1.03)	1.00 (0.98, 1.02)	1.00 (0.97, 1.03)
4 (highest)	0.98 (0.94, 1.01)	0.99 (0.96, 1.01)	1.00 (0.98, 1.02)	1.00 (0.97, 1.03)
Living arrangement				
Household of >1	1.00	1.00	1.00	1.00
Alone	1.01 (0.99, 1.03)	1.00 (0.98, 1.01)	1.01 (0.99, 1.02)	0.98 (0.96, 1.01)
Institution	**1.09 (1.04, 1.15)**	**1.07 (1.04, 1.10)**	**1.03 (1.01, 1.05)**	1.01 (0.98, 1.04)
Region				
Coast	1.00	1.00	1.00	1.00
South	1.02 (0.99, 1.05)	1.02 (1.00, 1.04)	1.01 (0.99, 1.03)	1.01 (0.98, 1.04)
Center	1.00 (0.97, 1.04)	1.02 (1.00, 1.05)	1.00 (0.98, 1.02)	1.00 (0.97, 1.03)
North	1.03 (0.99, 1.06)	1.02 (1.00, 1.05)	1.02 (1.00, 1.04)	1.01 (0.97, 1.04)
**Pre-existing health conditions**				
Dementia				
No	1.00	1.00	1.00	1.00
Yes	**1.07 (1.00, 1.15)**	**1.07 (1.04, 1.10)**	**1.04 (1.02, 1.05)**	1.01 (0.98, 1.03)
Mental health disorders				
No	1.00	1.00	1.00	1.00
Yes	**1.06 (1.02, 1.10)**	**1.04 (1.01, 1.07)**	1.02 (0.99, 1.05)	0.98 (0.93, 1.04)
Cancer				
No	1.00	1.00	1.00	1.00
Yes	**0.94 (0.91, 0.97)**	**0.94 (0.92, 0.96)**	**0.93 (0.91, 0.95)**	**0.94 (0.90, 0.98)**
Diseases of the nervous system				
No	1.00	1.00	1.00	1.00
Yes	**1.05 (1.01, 1.08)**	0.99 (0.97, 1.02)	0.99 (0.97, 1.02)	0.99 (0.94, 1.04)
Diabetes				
No	1.00	1.00	1.00	1.00
Yes	1.00 (0.97, 1.03)	1.02 (0.99, 1.04)	**1.02 (1.00, 1.04)**	1.00 (0.97, 1.04)
Circulatory				
No	1.00	1.00	1.00	1.00
Yes	0.99 (0.96, 1.01)	1.00 (0.99, 1.02)	1.00 (0.99, 1.02)	0.99 (0.97, 1.02)
Respiratory				
No	1.00	1.00	1.00	1.00
Yes	**1.03 (1.00, 1.06)**	**1.03 (1.01, 1.05)**	**1.02 (1.00, 1.04)**	1.01 (0.98, 1.04)
Alcohol related causes				
No	1.00	1.00	1.00	1.00
Yes	0.98 (0.92, 1.04)	0.98 (0.90, 1.07)	0.87 (0.73, 1.05)	1.19 (0.68, 2.08)
Accidents and violence				
No	1.00	1.00	1.00	1.00
Yes	0.99 (0.95, 1.03)	0.99 (0.96, 1.02)	1.00 (0.98, 1.02)	1.02 (0.98, 1.05)
Other than specified above				
No	1.00	1.00	1.00	1.00
Yes	1.00 (0.98, 1.02)	**0.96 (0.94, 0.98)**	0.99 (0.97, 1.00)	**0.97 (0.95, 0.99)**
Any health condition				
No	1.00	1.00	1.00	1.00
Yes	0.99 (0.96, 1.02)	1.00 (0.97, 1.02)	1.01 (0.99, 1.04)	0.97 (0.94, 1.00)

Note: Relative risks are the interaction terms of the winter indicator and the effect modifier. These show the relative differences in the effect of winter on mortality between effect modifier categories. Models are adjusted for sex, age, income, living arrangement, region, and year.

Table S4. Relative risks of excess winter mortality by selected individual characteristics, whether living in institutions or community. Finland 2000-2019, men and women aged 60 and over.

	**Winter-nonwinter interaction (95% CI)**	
	**In institution** n=156,851	**In community** n=668,073
Winter-nonwinter rate ratio	1.18 (1.16, 1.19)	1.11 (1.10, 1.12)
**Sociodemographic characteristics**		
Sex		
Men	1.00	1.00
Women	1.01 (0.99, 1.03)	**1.02 (1.01, 1.04)**
Age category		
60–69	1.00	1.00
70–79	1.02 (0.97, 1.07)	**1.03 (1.02, 1.05)**
80–89	1.01 (0.96, 1.06)	**1.07 (1.06, 1.09)**
90 and over	1.05 (1.00, 1.10)	**1.11 (1.09, 1.13)**
**Pre-existing health conditions**		
Dementia		
No	1.00	1.00
Yes	1.00 (0.98, 1.02)	**1.06 (1.05, 1.08)**
Respiratory		
No	1.00	1.00
Yes	1.01 (0.98, 1.04)	**1.03 (1.01, 1.04)**

Note: Relative risks are the interaction terms of the winter indicator and the effect modifier. These show the relative differences in the effect of winter on mortality between effect modifier categories. Models are adjusted for sex, age, income, living arrangement, region, and year.

Suulamo U, *et al. BMJ Open* 2024; 14:e079471. doi: 10.1136/bmjopen-2023-079471
