# Supplementary material for: Excess winter mortality in Finland, 1971–2019: a register-based study on long-term trends and effect modification by sociodemographic characteristics and pre-existing health conditions
Source: BMJ Open. 2024 Feb 2;14(2):e079471. doi: 10.1136/bmjopen-2023-079471 (PMC10840061; doi:10.1136/bmjopen-2023-079471)
Supplement: Author's manuscript [file bmjopen-2023-079471.draft_revisions.pdf]

BMJ Open

BMJ Open is committed to open peer review. As part of this commitment we make the peer review history of every article we publish publicly available.

When an article is published we post the peer reviewers' comments and the authors' responses online. We also post the versions of the paper that were used during peer review. These are the versions that the peer review comments apply to.

The versions of the paper that follow are the versions that were submitted during the peer review process. They are not the versions of record or the final published versions. They should not be cited or distributed as the published version of this manuscript.

BMJ Open is an open access journal and the full, final, typeset and author-corrected version of record of the manuscript is available on our site with no access controls, subscription charges or pay-per-view fees (http://bmjopen.bmj.com).

If you have any questions on BMJ Open's open peer review process please email info.bmjopen@bmj.com

BMJ Open

Excess winter mortality in Finland 1971-2019 – a prospective register-based study on long-term trends and effect modification by sociodemographic characteristics and pre-existing health conditions

Journal:	*BMJ Open*
Manuscript ID	bmjopen-2023-079471
Article Type:	Original research
Date Submitted by the Author:	01-Sep-2023

Complete List of Authors:	Suulamo, Ulla; University of Helsinki Faculty of Social Sciences<br>Remes, Hanna; University of Helsinki Faculty of Social Sciences<br>Tarkiainen , Lasse ; University of Helsinki Faculty of Social Sciences<br>Murphy, Michael; The London School of Economics and Political Science Department of Social Policy<br>Martikainen, Pekka; University of Helsinki Faculty of Social Sciences; Max-Planck-Institute for Demographic Research

Keywords:	Mortality, PUBLIC HEALTH, REGISTRIES, Risk Factors, Aged, EPIDEMIOLOGY

SCHOLARONE™
Manuscripts

I, the Submitting Author has the right to grant and does grant on behalf of all authors of the Work (as defined in the below author licence), an exclusive licence and/or a non-exclusive licence for contributions from authors who are: i) UK Crown employees; ii) where BMJ has agreed a CC-BY licence shall apply, and/or iii) in accordance with the terms applicable for US Federal Government officers or employees acting as part of their official duties; on a worldwide, perpetual, irrevocable, royalty-free basis to BMJ Publishing Group Ltd ("BMJ") its licensees and where the relevant Journal is co-owned by BMJ to the co-owners of the Journal, to publish the Work in this journal and any other BMJ products and to exploit all rights, as set out in our licence.

The Submitting Author accepts and understands that any supply made under these terms is made by BMJ to the Submitting Author unless you are acting as an employee on behalf of your employer or a postgraduate student of an affiliated institution which is paying any applicable article publishing charge ("APC") for Open Access articles. Where the Submitting Author wishes to make the Work available on an Open Access basis (and intends to pay the relevant APC), the terms of reuse of such Open Access shall be governed by a Creative Commons licence – details of these licences and which Creative Commons licence will apply to this Work are set out in our licence referred to above.

Other than as permitted in any relevant BMJ Author's Self Archiving Policies, I confirm this Work has not been accepted for publication elsewhere, is not being considered for publication elsewhere and does not duplicate material already published. I confirm all authors consent to publication of this Work and authorise the granting of this licence.

For peer review only

Original research

Excess winter mortality in Finland 1971-2019 – a prospective register-based study on long-term trends and effect modification by sociodemographic characteristics and pre-existing health conditions

Ulla Suulamo,[1,2] Hanna Remes,[1] Lasse Tarkiainen,[1] Mike Murphy,[3] and Pekka Martikainen[1,4,5]

Author Information

[1]Population Research Unit, Faculty of Social Sciences, University of Helsinki, Helsinki, Finland

[2]International Max Planck Research School for Population, Health and Data Science, Rostock, Germany

[3]Department of Social Policy, London School of Economics and Political Science, London, United Kingdom

[4]Max Planck Institute for Demographic Research, Rostock, Germany

[5]Max Planck – University of Helsinki Center for Social Inequalities in Population Health, Helsinki, Finland

Correspondence to: Ulla Suulamo, Population Research Unit, Faculty of Social Sciences, University of Helsinki, PO Box 42 (Unioninkatu 33), FIN-00014 Helsinki, Finland; ulla.suulamo@helsinki.fi

For peer review only

ABSTRACT

Objectives Excess winter mortality is a well-established phenomenon across the developed world. However, whether individual-level factors increase vulnerability to the effects of winter remains inadequately examined. Our aim was to assess long-term trends in excess winter mortality in Finland and estimate the effect of sociodemographic and health characteristics on the risk of winter death.

Design Prospective nationwide register study.

Setting Finland.

Participants Population aged 60 years and over resident in Finland in 1971-2019.

Outcome measures Age-adjusted winter and nonwinter death rates, winter-to-nonwinter rate ratios and relative risks.

Results We found a decreasing trend in the relative winter excess mortality over five decades and a drop in the series around 2000. In 2000-19 winter mortality rates for men and women were 11% and 14% higher than expected based on nonwinter rates. The relative risk of winter death increased with age but did not vary by income. Compared with those living with at least one other person, individuals in institutions had a higher relative risk (1.07, 95% CI 1.05–1.08). Most pre-existing health conditions did not predict winter death, but persons with dementia emerged at greater relative risk (1.06 [1.04–1.07]).

Conclusions Although winter mortality seems to affect frail people more strongly – those of advanced age, living in institutions and with dementia – there is an increased risk even beyond the more vulnerable groups. Protection of high-risk groups should be complemented with population-level preventive measures.

Keywords: Excess Mortality; Trends; Effect Modification; Risk Factors

Strengths and limitations of this study

- We used full-coverage high-quality, population-level register data spanning nearly 50 years with minimal attrition and no recall bias.
- The use of register data allowed us to examine risk factors at the individual level.
- We used data on healthcare visits and reimbursement for medicine costs to examine whether those with pre-existing health conditions are particularly vulnerable.
- The use of the underlying death cause may underestimate the total impact of respiratory infections and influenza-like illnesses on excess winter mortality.

For peer review only - http://bmjopen.bmj.com/site/about/guidelines.xhtml

INTRODUCTION

In temperate climates, mortality rates tend to be higher in winter compared to other times of the year. Although studies on long-term trends indicate that this winter excess has weakened over time,[1–4] it remains a significant public health burden in many high-income countries, with winter increases in deaths of up to 28 per cent.[5] This burden – likely driven both by the direct effects of winter conditions on the most frail individuals, as well as increased seasonal pressure on the health care system and knock-on effects on other patients – may even grow greater in ageing populations, considering that the risk of winter death increases with age and is highest among the oldest-old.[6,7]

The increased risk of dying in winter is attributed to multiple factors, most importantly influenza-like communicable illnesses[8,9] and low temperatures.[10] Consistent with these proximate risk factors, a winter peak in mortality is apparent for most major causes of death, but the largest seasonal fluctuations are seen for circulatory and respiratory diseases.[11] While such external effects drive the overall seasonal pattern, several non-seasonal risk factors have been found to importantly modify an individual's vulnerability to the effects of winter. Previous evidence is clear in relation to age: excess winter mortality especially affects older people, gradually increasing with age.[12] Besides age, factors such as low income, inadequate housing conditions and fuel poverty[13] have been associated with increased adverse health outcomes in winter, especially in countries with thermally inefficient housing stocks such as Portugal[14] and UK.[15] Although evidence is limited, living arrangement is another potentially important factor in differential mortality, and some previous findings suggest a higher risk of excess winter mortality for those living alone[12] or in care homes.[16–18]

The importance of non-seasonal risk factors is further demonstrated by cross-country comparisons, which suggest that absolute winter temperatures matter little, as countries with milder winters generally experience much greater wintertime excess than countries with colder climates.[5,19] These somewhat counterintuitive findings have led investigators to suggest that populations' ability to protect themselves is what ultimately determines the extent of winter mortality,[20] and to assume that winter excess deaths are, to an important degree, preventable.[5,7,21]

Despite the assumed avoidable character of a substantial fraction of excess winter mortality, uncertainty still exists whether the winter excess mainly affects frail individuals who are at greater risk of dying soon in any case. Those with pre-existing health conditions are often considered particularly vulnerable, but empirical evidence to confirm this is scarce. One previous study[22] assessing the determinants of vulnerability to excess winter mortality that took account of previous health found evidence for an increased risk of winter death only for those with a history of respiratory illness but not for those with other conditions. Addressing whether excess winter mortality mainly affects the frail remains thus an important area of further inquiry with relevant policy implications: identifying those most affected may help evaluate how preventable these excess deaths are and guide the development of targeted prevention measures.

For peer review only - http://bmjopen.bmj.com/site/about/guidelines.xhtml

In international comparison, northern European countries with relatively cold winters show lower levels of excess winter mortality which has been interpreted to arise from a combination of socioeconomic, housing, and behavioural factors.[19] Despite lower levels of excess, the winter period is nevertheless acknowledged to pose a range of health challenges,[23] and population ageing makes it likely that these challenges will become more pressing. Little is, however, known about how excess winter mortality differs by individual-level social and health characteristics in a societal context with high-quality housing at all income levels and universal access to health care services. Using total population individual-level data, we examined long-term trends in excess winter mortality in Finland at ages 60 and above in 1971–2019 and assessed differences in the winter excess by cause of death and the effect of sociodemographic characteristics and pre-existing health conditions in 2000–2019.

For peer review only

METHODS

Data

The study was based on individual-level data for the total population of Finland from August 1971 to July 2019. This data was drawn from censuses and population registers that Statistics Finland has linked to cause-of-death records and health care registers using personal identification numbers. All data was collected for routine administrative purposes and, therefore, public involvement was not feasible. For 1971-1987 sociodemographic information was collected from the end-of-year census (1970/1975/1980/1985) and from the year 1988 onwards from the end-of-year population records. We restricted the analysis to individuals 60 years and over in 1971-2019.

We defined winter as December-March following previous studies in the northern hemisphere.[13] These months also meet the definition of thermal winter in Finland with daily mean temperatures below 0°C (32°F). On average, in central and southern Finland, where most of the population resides, thermal winter begins around late November and ends in late March. Nonwinter months were defined as the four months on each side of this winter period (August-November, April-July). Following this definition, our study period to analyse trends over time ran from August 1971 to July 2019, 48 years in total.

For the more recent 19-year period August 2000 – July 2019, we assessed whether and how excess winter mortality was modified by sex, age, living arrangement, income, region, and pre-existing health conditions. Age was categorized into groups 60-69, 70-79, 80-89 and 90 and over. Living arrangement distinguished between those living in households with at least one other person, alone or in institutions. Household disposable income was categorized into quartiles in the population aged 60 and over and adjusted for the household composition.[24] For persons living in institutions, we used personal disposable income. Information on region of residence (n=19) was aggregated into four larger regions (coastal, southern, central, and northern) according to average winter

For peer review only - http://bmjopen.bmj.com/site/about/guidelines.xhtml

temperatures. Living arrangement, income and region were measured at the end of the preceding calendar year, whereas age corresponded to attained age in July of each year.

We used ten dichotomous indicators of pre-existing health conditions: dementia, mental disorders, diseases of the nervous system, diabetes, diseases of the circulatory system, diseases of the respiratory system, alcohol-related diseases, injuries due to accidents and violence, and other health conditions. These conditions were identified from two sources covering the years 1999-2017: the health care register of the Finnish Institute for Health and Welfare and the medication reimbursement register of the Social Insurance Institution of Finland. We included data on inpatient and specialized outpatient care episodes that occurred during the preceding calendar year and for which the aforementioned conditions were noted as the primary discharge diagnoses based on the International Classification of Diseases [ICD] 10th Revision. Information on the right to special reimbursement for drug costs due to diagnosed chronic medical conditions during the preceding calendar year was collected from the medication reimbursement register of the Social Insurance Institution of Finland following their disease and medication classification. For exact coding of care episodes and reimbursement categories see Table S1 in the Supplemental Material. A pre-existing health condition was considered present if there was a record in either of the two registers and one individual could contribute to several categories.

We considered all-cause mortality as well as mortality from diseases of the circulatory system (International Classification of Diseases ICD 10th Revision codes I00-I425, I427-I99), diseases of the respiratory system (J00-J64, J66-J99), dementia (F01, F03, G30, R54), other internal causes and external (V01-X44, X46-Y89) causes. The cause of death coding was based on Statistics Finland's time series cause of death classification, which harmonizes data across different revisions of the ICD.

Statistical analysis

To obtain person-days at risk for each winter and nonwinter period, we created an expanded dataset by splitting each year on April 1st and December 1st. Deaths were allocated to the appropriate winter and nonwinter periods using exact death dates.

To assess trends over time, we collapsed the expanded dataset by summing the death and person-time counts over sex, age, year, and winter and calculated and plotted age-adjusted annual winter and nonwinter death rates with 95% confidence intervals (CI) in 1971-2019 using the age structure of the entire period as the standard population. Both absolute and relative differences between winter and nonwinter rates were computed. To identify shifts in the trends, we explored the presence and location of breakpoints in the series of absolute and relative differences. We applied segmented linear regression analysis with one break on the cumulative values using the "segmented" package[25] in R software version 4.2.2 (R Core Team).

For peer review only - http://bmjopen.bmj.com/site/about/guidelines.xhtml

To study the extent to which individual characteristics modified the risk of excess winter mortality, we focused on the more recent period from August 2000 to July 2019. We assessed effect modification using Poisson regression with interactions between the winter indicator and the covariates. Each potential effect modifier was studied separately, adjusting the models for all the other covariates.

RESULTS

Over the study period from August 1971 through July 2019, 1,902,824 deaths were recorded, of which 36% occurred during winter.

The age-adjusted winter death rate fell from 95 deaths per 1,000 person-years among men and 66 among women in 1971 to 37 and 23 in 2018, respectively (Figure 1). The nonwinter death rate was consistently below that of winter throughout the observation period and decreased from 78 to 33 among men and from 53 to 20 among women.

Absolute and relative differences between winter and nonwinter mortality (Figure 2) showed considerable year-to-year fluctuations. Overall, mortality declined over time, as did the absolute difference between winter and nonwinter mortality rates - from an average of around 15 deaths per 1,000 person-years to below five. Relative differences fluctuated between a winter excess of 31% (women year 1975) and only 4% (men year 2013), showing a clear downward trend. Changes in absolute differences for women were somewhat smaller than for men, and vice versa for relative differences, but peaks and troughs were in the same years for both sexes.

Based on the figures, towards the end of the study period, the values of both absolute and relative differences oscillated at lower levels, and the year-to-year variation was less pronounced. A breakpoint analysis indicated a change in the series around the year 2000 for both absolute and relative differences. The drop in the mean rate difference after the estimated break was nearly 50%, whereas for rate ratios, the drop was smaller.

Descriptive information about the study population at the ends of the more recent period 2000-2019 shows population ageing and an increasing share of men (Table 1). Most health conditions have become more common, except for diseases of the circulatory system. The percentage of individuals recorded with dementia increased from 1.4 to 3.7%. However, the proportion with no health care records during the previous calendar year remained at one third. Living arrangements were stable, with around 3% living in institutions, one-third alone, and the rest with at least one other person.

For peer review only - http://bmjopen.bmj.com/site/about/guidelines.xhtml

Table 1. Characteristics of the study population in 2000/01 and 2018/19.

	2000/01 (%)	2018/19 (%)
**Sociodemographic characteristics**		
Mean age (sd)	71.2 (8.1)	71.6 (8.4)
Sex		
Men	40.8	45.0
Women	59.2	55.0
Age category		
60–69	47.2	46.8
70–79	35.9	34.0
80–89	14.7	15.9
90 and over	2.1	3.2
Income quartile		
1 (lowest)	25.0	25.0
2	25.0	25.0
3	25.0	25.0
4 (highest)	25.0	25.0
Living arrangement		
Household of >1	63.3	63.2
Alone	33.4	33.8
Institution	3.3	3.0
Large regions		
Coast	36.4	38.7
South	29.0	27.8
Center	19.2	17.9
North	15.5	15.6
**Pre-existing health conditions**		
Dementia	1.4	3.7
Mental health disorders	3.7	3.8
Diseases of the nervous system	4.4	6.7
Diabetes	7.4	15.0
Diseases of the circulatory system	44.4	35.5
Diseases of the respiratory system	9.4	11.4
Alcohol related diseases	0.3	0.5
Accidents and violence	4.4	6.4
Other	29.8	36.1
None	34.2	33.2
Total	100.0	100.0
N	1,035,643	1,559,165

In 2000-2019 all-cause mortality during winter occurred at an age- and sex-adjusted rate of 49.8 per 1,000 person-years among men, and 31.4 per 1,000 person-years among women, which was 11% and 14% higher than in the nonwinter period, respectively (Table 2). The winter excess varied by cause of death. It was observed for all diseases, except for external causes among men, and was strongest for respiratory disease deaths. For men, 26% and for women, 40% more deaths due to a respiratory cause occurred during the winter period than expected based on the nonwinter average. Those who died from dementia showed the second highest winter excess (men 21%, women 22%), exceeding that of circulatory disease deaths (men 13%, women 14%).

For peer review only - http://bmjopen.bmj.com/site/about/guidelines.xhtml

Table 2. Age- and sex-adjusted death rates in winter and nonwinter periods and rate ratios, by cause of death and sex. Finland 2000-2019, ages 60 and over.

	Men				Women			
	Deaths	Rate per 1,000 person years		Winter-nonwinter rate ratio	Deaths	Rate per 1,000 person years		Winter-nonwinter rate ratio
		Winter	Nonwinter			Winter	Nonwinter	
Circulatory	160,087	21.7	19.3	1.13 (1.12, 1.14)	190,933	13.5	11.8	1.14 (1.13, 1.15)
Respiratory	24,943	3.5	2.8	1.26 (1.23, 1.30)	18,236	1.5	1.1	1.40 (1.35, 1.44)
Dementia	36,659	6.0	4.9	1.21 (1.18, 1.24)	82,108	5.7	4.7	1.22 (1.20, 1.24)
Other internal	132,285	14.9	14.3	1.04 (1.03, 1.06)	131,892	9.4	8.9	1.06 (1.05, 1.07)
External	30,191	3.3	3.2	1.01 (0.99, 1.04)	17,590	1.3	1.2	1.06 (1.03, 1.10)
All causes	384,165	49.5	44.8	1.11 (1.10, 1.11)	440,759	31.4	27.6	1.14 (1.13, 1.14)

Table 3 shows winter and nonwinter death rates and their adjusted rate ratios for different categories of potential effect modifiers, and the adjusted ratio of these winter-nonwinter ratios relative to the ratio of the baseline reference group. The adjusted risk of dying in winter increased with age and was slightly higher for women (1.14) than men (1.11), translating to a relative difference of RR=1.03, 95% CI 1.02–1.04. There was no evidence that the risk of excess winter death varied by income and only weak evidence of differences between regions. The risk of dying in winter was relatively higher for persons living in institutions (RR=1.07, 95% CI 1.05–1.08), but those who lived alone did not differ much from those living with at least one other person. Overall, simultaneous adjustment for all covariates had very minor effects on the results.

All analysed categories of pre-existing health conditions were associated with higher risk of death during both winter and nonwinter periods. However, their effect on the relative risk of winter mortality was generally modest. The most substantial effect modification was found for persons with a history of dementia with an adjusted winter-to-nonwinter rate ratio of 1.17 (95% CI 1.16–1.19) and a risk ratio of 1.06 (95% CI 1.04–1.07) when comparing those with dementia to those without. Other categories of health conditions that seemed to somewhat increase the risk of dying in winter included diseases of the respiratory system (RR=1.02, 95% CI 1.02-1.03) and mental disorders (RR=1.02, 95% CI 1.00-1.04. For alcohol-related causes the effect was the other way around with a rate ratio of 0.93 (95% CI 0.89–0.98).

Age-category-specific results (Table S3) showed that while winter excess mortality increased with age, the effects of the analysed sociodemographic and health characteristics were mostly similar. Effect modification was, however, more apparent among the younger age categories, whereas among 90-year-olds, no effect modification was observed.

For peer review only - http://bmjopen.bmj.com/site/about/guidelines.xhtml

Table 3. Death rates in winter and nonwinter periods, rate ratios, and relative differences in excess winter mortality by individual characteristics. Finland 2000-2019, men and women aged 60 and over.

	Rate per 1000 person years*		Winter-nonwinter rate ratio (95% CI)[a]	Winter-nonwinter interaction (95% CI)[b]
	Winter (n=295,236)	Nonwinter (n=529,688)		
**Sociodemographic characteristics**				
All	37.8	33.7	1.12 (1.12, 1.13)	
Sex				
Men	49.5	44.8	1.11 (1.10, 1.11)	1.00
Women	31.4	27.6	1.14 (1.13, 1.14)	**1.03 (1.02, 1.04)**
Age category				
60–69	11.9	11.2	1.06 (1.05, 1.07)	1.00
70–79	30.7	27.9	1.10 (1.09, 1.11)	**1.04 (1.02, 1.05)**
80–89	94.6	83.2	1.14 (1.13, 1.15)	**1.07 (1.06, 1.09)**
90 and over	269.8	228.0	1.19 (1.17, 1.20)	**1.12 (1.10, 1.13)**
Income quartile				
1 (lowest)	41.5	37.2	1.12 (1.11, 1.13)	1.00
2	38.5	34.3	1.12 (1.11, 1.13)	1.00 (0.99, 1.02)
3	37.9	33.7	1.12 (1.11, 1.13)	1.01 (0.99, 1.02)
4 (highest)	32.9	29.2	1.13 (1.11, 1.14)	1.01 (0.99, 1.02)
Living arrangement				
Household of >1	31.0	28.0	1.10 (1.10, 1.11)	1.00
Alone	34.3	30.6	1.12 (1.11, 1.13)	**1.01 (1.00, 1.02)**
Institution	110.1	93.6	1.18 (1.16, 1.19)	**1.07 (1.05, 1.08)**
Region				
Coast	36.8	33.1	1.11 (1.10, 1.12)	1.00
South	37.5	33.2	1.13 (1.12, 1.14)	**1.02 (1.00, 1.03)**
Center	38.9	34.8	1.12 (1.11, 1.13)	1.01 (0.99, 1.02)
North	39.3	34.7	1.13 (1.12, 1.15)	**1.02 (1.01, 1.03)**
**Pre-existing health conditions**				
Dementia				
No	34.0	30.6	1.11 (1.11, 1.12)	1.00
Yes	78.9	67.2	1.17 (1.16, 1.19)	**1.06 (1.04, 1.07)**
Mental health disorders				
No	36.5	32.6	1.12 (1.12, 1.13)	1.00
Yes	69.6	60.9	1.14 (1.12, 1.16)	**1.02 (1.00, 1.04)**
Diseases of the nervous system				
No	36.1	32.2	1.12 (1.12, 1.13)	1.00
Yes	65.6	58.7	1.12 (1.10, 1.13)	0.99 (0.98, 1.01)
Diabetes				
No	35.2	31.4	1.12 (1.12, 1.13)	1.00
Yes	57.0	50.5	1.13 (1.12, 1.14)	1.01 (1.00, 1.02)
Circulatory				
No	29.9	26.8	1.12 (1.11, 1.13)	1.00
Yes	45.5	40.4	1.13 (1.12, 1.13)	1.01 (1.00, 1.02)
Respiratory				
No	34.7	31.0	1.12 (1.11, 1.12)	1.00
Yes	61.2	53.6	1.14 (1.13, 1.15)	**1.02 (1.01, 1.03)**
Alcohol related				
No	37.5	33.4	1.12 (1.12, 1.13)	1.00
Yes	169.7	161.8	1.05 (1.00, 1.10)	**0.93 (0.89, 0.98)**
Accidents and violence				
No	36.4	32.5	1.12 (1.12, 1.13)	1.00

For peer review only - http://bmjopen.bmj.com/site/about/guidelines.xhtml

Yes	56.0	49.7	1.13 (1.11, 1.14)	1.00 (0.99, 1.02)
Other				
No	32.7	28.7	1.14 (1.13, 1.15)	1.00
Yes	45.8	41.5	1.10 (1.10, 1.11)	**0.97 (0.96, 0.98)**
None				
No	43.8	39.0	1.12 (1.12, 1.13)	1.00
Yes	19.3	17.3	1.12 (1.11, 1.13)	1.00 (0.98, 1.01)

[a]Adjusted for age (excl. age category)

[b]Adjusted for sex, age, income, living arrangement, region, year

DISCUSSION

Main findings

With individual-level total population data covering nearly half a century, we examined long-term trends in excess winter mortality in Finland and assessed how individual-level characteristics may modify the risk of dying in winter. We showed that both winter and nonwinter death rates steadily declined in 1971-2019, but winter rates remained above nonwinter rates through the period. All-cause mortality was 11% and 14% higher during winter compared to nonwinter months in 2000-19 for men and women, respectively, with highest excess in respiratory and dementia related causes of death. We found modest effect modification by sociodemographic characteristics and pre-existing health conditions. Advanced age, living in institutions, and dementia increased the risk of winter mortality, while income level and most health conditions made no difference.

Interpretation of the results

Previous research has documented downward trends in excess winter mortality during most of the 20th century.[2,3,26,27] The declining trend has been attributed to general socioeconomic progress, improved housing conditions and healthcare systems. According to previous research, differences between winter and summer mortality started to decline relatively early in Finland, in the 1930s,[28] and a slowly decreasing trend was also reported during the latter part of the century, until 1995.[29] Although not directly comparable to ours because of different definition of the winter excess (deaths in September-March compared to the month of lowest mortality) and age ranges considered, these previous results do align with our findings. In addition, with our results extending over 20 years further, we report a clear attenuation in the year-to-year variability during the last two decades and a downturn in the rate ratios around the year 2000.

Influenza-like illnesses are an important contributor to excess winter mortality, some studies showing that it is especially these – and not cold severity – that nowadays explain the year-to-year variations in the winter excess.[30,31] There are at least two influenza-related factors that may have contributed to the smaller winter mortality fluctuations observed. During the first decade of the 21st century, seasonal influenza activity and related mortality were relatively low compared to previous

For peer review only - http://bmjopen.bmj.com/site/about/guidelines.xhtml

decades.[32] Also, since 2002, influenza vaccines have been administered as part of the national vaccination programme in Finland for all those aged 65 years and older. Vaccination is considered the best available intervention to prevent influenza illness, and some previous studies have reported that the influenza vaccine significantly reduces mortality.[33,34] Although more research is needed as the mortality benefits of influenza vaccine, especially among the older population, have been questioned,[35,36] our results are consistent with the idea that both lower influenza activity of recent decades and introduction of vaccinations may have reduced excess winter mortality peaks.

Age is known to play an important role for excess winter mortality,[12,37] and of all the individual-level effect modifiers included in our analysis, older age showed the strongest and clearest influence. The increasing risk with age is commonly interpreted as a reflection of increasing frailty and vulnerability towards adverse environmental stresses as age advances.[38] Accordingly, we found that persons living in institutions were likelier to die in winter. As previous studies with similar findings from France,[16] Netherlands[17] and England and Wales[18] note, those living in institutions are rarely exposed to outside weather and cold. However, they are likely to spend more time indoors within confined environments, which may increase the spread of infections.[39] Furthermore, the prevalence of frailty is considerably high among older adults living in residential care,[40] indicating their increased risk of mortality and vulnerability to external stressors.[41]

Apart from frailty associated with advanced age, it is generally assumed that excess winter mortality most importantly affects persons with pre-existing health conditions. Nevertheless, previous literature approaching the topic by including information about the prior health of those dying in winter is scarce. In general, our findings showed very weak or no evidence for increased risk for dying in winter among those with pre-existing conditions. Only persons with dementia emerged as being at greater risk, a finding in line with few previous studies. Our results concerning dementia mortality also support these findings, as those who died from dementia showed the second-highest winter excess of around 21%. Previously, Liddell and co-authors[42] have also reported almost a third more dementia deaths in winter compared to nonwinter times in the UK. While a tendency to attribute deaths to the underlying chronic condition instead of a respiratory infection could explain some of the excess, another possible interpretation is that those with dementia have difficulties in communicating changes in their health status to relatives and care staff. In a study on susceptibility to cold, Zanobetti and co-authors[43] found that in the US, persons with previous admission for dementia were at greater risk of dying on extremely cold days and it has been speculated that this may relate to reduced perception of and protection from cold.[44]

By contrast, the risk of dying in winter was not significantly increased for those with other pre-existing health conditions. Not even those with respiratory or circulatory conditions seemed significantly more vulnerable, although persons with these conditions are traditionally assumed to face a particularly high risk of death in winter. This common assumption may partly arise from considering stated causes of death as pre-existing conditions and thus interpreting the findings that

For peer review only - http://bmjopen.bmj.com/site/about/guidelines.xhtml

show high excess winter mortality for cardiovascular and respiratory causes as an indication that individuals with pre-existing cardiovascular or respiratory disease are more susceptible to the adverse effects of winter. We also found the highest excess winter mortality in deaths due to diseases of the respiratory system and a somewhat lesser excess risk in deaths due to circulatory diseases, but a similar excess risk did not apply to people diagnosed with these conditions in the preceding year. Previous evidence on the effect of pre-existing health conditions – primarily based on studies on cold-related mortality and not on excess winter mortality specifically – is conflicting. For example, while Wilkinson and co-authors found that pre-existing respiratory disease increased the risk of a winter death, and Schwartz[45] reported elevated risks on cold days for persons with chronic obstructive pulmonary disease, other empirical studies have found no higher risks than average[46] and even lower than average risks have been reported.[43] Similarly, for cardiovascular conditions, there is some evidence both for[47,48] and against[45,49,50] an increased risk of winter death. One possible explanation for not detecting higher risks for most of the pre-existing health conditions – including cardiovascular and respiratory diseases – may be that persons with a diagnosis and previous healthcare contact are the ones who receive most of the preventive measures. If this is true, it suggests there is room for prevention among the apparently healthy population.

While there is a well-known link between socioeconomic position and overall mortality,[51] we found no evidence to suggest a socioeconomic gradient in vulnerability to excess winter mortality when measured by income. Instead, the increase in mortality in winter was similar in all income categories. Previous studies regarding the association between socioeconomic factors and excess winter mortality have provided conflicting results.[13] Hales and co-authors,[52] for example, found that the risk of winter death in New Zealand was higher for those in the lowest income tertile compared to those in the highest tertile. Many of the proposed mechanisms through which lower socioeconomic position and income may increase the risk of winter death are linked to housing conditions and factors such as central heating and fuel poverty.[13] However, it has also been noted that low socio-economic position does not always go hand in hand with the poorest quality housing, as in some countries, e.g. the UK, social housing may even have better than average energy efficiency characteristics.[22] In Finland, housing is generally of high quality, and conditions are relatively good at all income levels. Homes and buildings are well insulated, and most households can keep their homes adequately warm regardless of low outside temperatures,[53] which probably partly explains why no income gradient was observed in our study. Similar conclusions were drawn from a previous study in another Nordic country, Denmark.[12]

Methodological considerations

Our study is based on full-coverage high-quality, population-level register data with minimal attrition and no underreporting or recall bias. The use of register data allowed us to examine risk factors at the individual level, a key limitation in much of the previous work on excess winter

For peer review only - http://bmjopen.bmj.com/site/about/guidelines.xhtml

mortality. Nevertheless, we recognize that identifying persons with pre-existing health conditions based on healthcare visits and reimbursement for medicine costs may present some limitations as these records may reflect differences in health-seeking behaviour. Another issue relates to the timing of the measurement. If we measured health too close to death, we might be capturing the beneficial effect of being monitored by healthcare professionals or the results could be biased by a survivor effect. Our results are based on measuring health during the preceding calendar year (a 7-month "quarantine" period from January-July). We replicated the analysis measuring health closer (no quarantine period) and further back (extended quarantine period). Results, however, remained unchanged, which makes us confident to argue that the timing of the measurement is not driving the small effects.

The described cause-specific findings based on the underlying death cause may significantly underestimate the total impact of respiratory infections and influenza-like illnesses on excess winter mortality.[21] Deaths are rarely coded as influenza in death certificates[54] but instead attributed to chronic diseases, especially among the older population. Nevertheless, respiratory infections are important triggers of, for instance, cardiovascular problems.[55] To better understand the mechanisms behind excess winter mortality, future research should consider information on contributory causes of death to assess the indirect triggering effect of respiratory diseases.

Conclusions and implications

In conclusion, with high-quality individual-level total population data, we showed that winter mortality has declined since 1971, and particularly after year 2000, but remained above nonwinter rates throughout. In particular, we provide robust evidence that those with advanced age, living in institutions, and dementia had a particularly high risk of winter mortality. The subgroup differences were relatively small overall, suggesting an increased risk of winter death even beyond the more vulnerable subgroups. In the context of population ageing and increasing numbers of individuals surviving into ages where excess winter mortality reaches high levels, public health action to protect high-risk groups may have wider population-level benefits.

Contributors All authors contributed to the study conception and design. US prepared the data, conducted the analyses, conducted the literature review, and wrote the first version of the manuscript. All authors critically revised, commented, and edited the draft versions of the manuscript for important intellectual content and approved its final version.

Funding US was supported by Jenny and Antti Wihuri Foundation (grant numbers 00210374 and 0022038). PM was supported by the European Research Council under the European Union's Horizon 2020 research and innovation programme (grant agreement number 101019329), the Strategic Research Council (SRC) within the Academy of Finland grants for ACElife (352543-352572) and LIFECON (308247), and grants to the Max Planck – University of Helsinki Center

For peer review only - http://bmjopen.bmj.com/site/about/guidelines.xhtml

from the Jane and Aatos Erkko Foundation, the Max Planck Society, University of Helsinki, and Cities of Helsinki, Vantaa and Espoo.

Disclaimer The study does not necessarily reflect the Commission's views and in no way anticipates the Commission's future policy in this area. The funders had no role in the study design, data collection and analysis, decision to publish, or preparation of the manuscript.

Competing interests None declared

Patient consent Not required.

Ethics approval The use of the anonymized register data has been approved by Statistics Finland's Ethics Board. The register data are originally collected for administrative and statistical purposes. No informed consent is required for register-based studies in Finland.

Data sharing Data are not publicly available. Due to data protection regulations of the register-holders, the authors are unable to make the data available to third parties. In order to use the data, researchers and research institutions need to apply for a license that authorizes its use for research purposes. Access to the data can be requested by contacting the register holders.

Acknowledgements US gratefully acknowledges the resources provided by the International Max Planck Research School for Population, Health and Data Science (IMPRS-PHDS).

For peer review only

REFERENCES

1. Almendra R, Perelman J, Vasconcelos J, et al. Excess winter mortality and morbidity before, during, and after the Great Recession: the Portuguese case. Int J Biometeorol 2019;63:873–883. doi:10.1007/s00484-019-01700-6

2. Kunst AE, Looman CW, Mackenbach JP. The decline in winter excess mortality in The Netherlands. International Journal of Epidemiology 1991;20:971–977. doi:10.1093/ije/20.4.971

3. Ledberg A. A large decrease in the magnitude of seasonal fluctuations in mortality among elderly explains part of the increase in longevity in Sweden during 20th century. BMC Public Health 2020;20:1674. doi:10.1186/s12889-020-09749-4

4. Lerchl A. Changes in the seasonality of mortality in Germany from 1946 to 1995: the role of temperature. Int J Biometeorol 1998;42:84–88. doi:10.1007/s004840050089

5. Fowler T, Southgate RJ, Waite T, et al. Excess winter deaths in Europe: a multi-country descriptive analysis. Eur J Public Health 2015;25:339–345. doi:10.1093/eurpub/cku073

For peer review only - http://bmjopen.bmj.com/site/about/guidelines.xhtml

6. Parks RM, Bennett JE, Foreman KJ, et al. National and regional seasonal dynamics of all-cause and cause-specific mortality in the USA from 1980 to 2016. Elife 2018;7:e35500. doi:10.7554/eLife.35500

7. Davie GS, Baker MG, Hales S, et al. Trends and determinants of excess winter mortality in New Zealand: 1980 to 2000. BMC Public Health 2007;7:263–272. doi:10.1186/1471-2458-7-263

8. Reichert TA, Simonsen L, Sharma A, et al. Influenza and the Winter Increase in Mortality in the United States, 1959–1999. American Journal of Epidemiology 2004;160:492–502. doi:10.1093/aje/kwh227

9. Vestergaard LS, Nielsen J, Krause TG, et al. Excess all-cause and influenza-attributable mortality in Europe, December 2016 to February 2017. Eurosurveillance 2017;22:30506: http://dx.doi.org/10.2807/1560-7917.ES.2017.22.14.30506

10. Gasparrini A, Guo Y, Hashizume M, et al. Mortality risk attributable to high and low ambient temperature: a multicountry observational study. Lancet 2015;386:369–375. doi:10.1016/S0140-6736(14)62114-0

11. Rau R, Bohk-Ewald C, Muszyńska MM, et al. Seasonality of Causes of Death. In: Rau, R, Bohk-Ewald, C, Muszyńska MM, et al. eds. Visualizing Mortality Dynamics in the Lexis Diagram. The Springer Series on Demographic Methods and Population Analysis. Springer Cham 2018:99–122.

12. Rau R. Seasonality in human mortality: a demographic approach. Berlin; New York: Springer 2007.

13. Tanner LM, Moffatt S, Milne EMG, et al. Socioeconomic and behavioural risk factors for adverse winter health and social outcomes in economically developed countries: a systematic review of quantitative observational studies. J Epidemiol Community Health 2013;67:1061–1067. doi:10.1136/jech-2013-202693

14. Almendra R, Santana P, Vasconcelos J. Evidence of social deprivation on the spatial patterns of excess winter mortality. Int J Public Health 2017;62:849–856. doi:10.1007/s00038-017-0964-7

15. Stacey D, Pritchard C. An ecological study of excess winter mortality in England and deprivation. Public Health 2016;141:207–209. doi:10.1016/j.puhe.2016.09.023

16. Phu Pin S, Golmard JL, Cotto E, et al. Excess Winter Mortality in France: Influence of Temperature, Influenza Like Illness, and Residential Care Status. Journal of the American Medical Directors Association 2012;13:309.e1-309.e7. doi:10.1016/j.jamda.2011.06.005

17. Rolden HJA, Rohling JHT, van Bodegom D, et al. Seasonal Variation in Mortality, Medical Care Expenditure and Institutionalization in Older People: Evidence from a Dutch Cohort of Older Health Insurance Clients. PLoS One 2015;10:e0143154. doi:10.1371/journal.pone.0143154

For peer review only

18. Hajat S, Kovats RS, Lachowycz K. Heat-related and cold-related deaths in England and Wales: who is at risk? Occup Environ Med 2006;64:93–100. doi:10.1136/oem.2006.029017

19. Healy JD. Excess winter mortality in Europe: a cross country analysis identifying key risk factors. Journal of Epidemiology and Community Health 2003;57:784–789. doi:10.1136/jech.57.10.784

20. Gemmell I. Seasonal variation in mortality in Scotland. International Journal of Epidemiology 2000;29:274–279. doi:10.1093/ije/29.2.274

21. Shkolnikov VM, Klimkin I, McKee M, et al. What should be the baseline when calculating excess mortality? New approaches suggest that we have underestimated the impact of the COVID-19 pandemic and previous winter peaks. SSM Popul Health 2022;18:101118. doi:10.1016/j.ssmph.2022.101118

22. Wilkinson P, Pattenden S, Armstrong B, et al. Vulnerability to winter mortality in elderly people in Britain: population based study. BMJ 2004;329:647. doi:10.1136/bmj.38167.589907.55

23. Reinikainen M, Uusaro A, Ruokonen E, et al. Excess mortality in winter in Finnish intensive care. Acta Anaesthesiol Scand 2006;50:706–711. doi:10.1111/j.1399-6576.2006.01041.x

24. OECD. Framework for integrated analysis. In: OECD Framework for Statistics on the Distribution of Household Income, Consumption and Wealth. OECD. 2013;171–192.

25. Muggeo VMR. segmented: An R Package to Fit Regression Models with Broken-Line Relationships. 2008.

26. Donaldson GC, Keatinge WR. Mortality related to cold weather in elderly people in southeast England, 1979-94. BMJ 1997;315:1055–1056. doi:10.1136/bmj.315.7115.1055

27. Ekamper P, Van Poppel F, Van Duin C, et al. 150 Years of temperature-related excess mortality in the Netherlands. Demographic Research 2009;21:385–426. doi:10.4054/DemRes.2009.21.14

28. Näyhä S. Short and Medium-Term Variations in Mortality in Finland: A study on cyclic variations, annual and weekly periods and certain irregular changes in mortality in Finland during the period 1868-1972. Scand J Soc Med Suppl. 1981;21:1-101. PMID:7022620

29. Näyhä S. Seasonal variation of deaths in Finland--is it still diminishing? Int J Circumpolar Health 2000;59: 182–7. PMID:11209665

30. von Klot S, Zanobetti A, Schwartz J. Influenza epidemics, seasonality, and the effects of cold weather on cardiac mortality. Environ Health 2012;11:74. doi:10.1186/1476-069X-11-74

31. Kinney PL, Schwartz J, Pascal M, et al. Winter season mortality: will climate warming bring benefits? Environ Res Lett 2015;10:064016. doi: 10.1088/1748-9326/10/6/064016

For peer review only - http://bmjopen.bmj.com/site/about/guidelines.xhtml

32. Jones RP. Excess winter mortality and stalling international improvements in life expectancy and mortality rates. Brit J Healthc Manage 2020;26:1–8. doi:10.12968/bjhc.2020.0020

33. Voordouw BCG, van der Linden PD, Simonian S, et al. Influenza vaccination in community-dwelling elderly: impact on mortality and influenza-associated morbidity. Arch Intern Med 2003;163:1089–1094. doi:10.1001/archinte.163.9.1089

34. Fireman B, Lee J, Lewis N, et al. Influenza vaccination and mortality: differentiating vaccine effects from bias. Am J Epidemiol 2009;170:650–656. doi:10.1093/aje/kwp173

35. Simonsen L, Taylor RJ, Viboud C, et al. Mortality benefits of influenza vaccination in elderly people: an ongoing controversy. Lancet Infect Dis 2007;7:658–666. doi:10.1016/S1473-3099(07)70236-0

36. Demicheli V, Jefferson T, Pietrantonj CD, et al. Vaccines for preventing influenza in the elderly. Cochrane Database Syst Rev: 2018;2(2):CD004876. doi: 10.1002/14651858.CD004876.pub4

37. Feinstein CA. Seasonality of deaths in the U.S. by age and cause. Demographic Research 2002;6:471–488. doi:10.4054/DemRes.2002.6.17

38. Robine J-M. When climate change encounters the revolution in adult longevity. Aging Clin Exp Res 2017;29:1073–1074. doi:10.1007/s40520-017-0839-z

39. Arbuthnott K, Hajat S, Heaviside C, et al. What is cold-related mortality? A multi-disciplinary perspective to inform climate change impact assessments. Environ Int 2018;121:119–129. doi:10.1016/j.envint.2018.08.053

40. Milte R, Petersen J, Boylan J, et al. Prevalence and determinants of physical frailty among people living in residential aged care facilities: a large-scale retrospective audit. BMC Geriatrics 2022;22:424. doi:10.1186/s12877-022-03101-8

41. Dent E, Ambagtsheer RC, Beilby J, et al. Frailty and Seasonality. J Nutr Health Aging 2020;24:547–549. doi:10.1007/s12603-020-1367-7

42. Liddell C, Morris C, Gray B, et al. Excess winter mortality associated with Alzheimer's Disease and related dementias in the UK: A case for energy justice. Energy Research & Social Science 2016;11:256–262. doi:10.1016/j.erss.2015.11.007

43. Zanobetti A, O'Neill MS, Gronlund CJ, et al. Susceptibility to Mortality in Weather Extremes: Effect Modification by Personal and Small-Area Characteristics. Epidemiology 2013;24:809–819. doi:10.1097/01.ede.0000434432.06765.91

For peer review only

44. Rocklöv J, Forsberg B, Ebi K, et al. Susceptibility to mortality related to temperature and heat and cold wave duration in the population of Stockholm County, Sweden. Global Health Action 2014;7:22737. doi:10.3402/gha.v7.22737

45. Schwartz J. Who is Sensitive to Extremes of Temperature? A Case-Only Analysis. Epidemiology 2005;16:67–72. doi:10.1097/01.ede.0000147114.25957.71

46. Tammes P, Sartini C, Preston I, et al. Use of primary care data to predict those most vulnerable to cold weather: a case-crossover analysis. Br J Gen Pract 2018;68:e146–e156. doi:10.3399/bjgp18X694829

47. Bhaskaran K, Hajat S, Haines A. et al. Short term effects of temperature on risk of myocardial infarction in England and Wales: time series regression analysis of the Myocardial Ischaemia National Audit Project (MINAP) registry. BMJ 2010;341:c3823. doi:10.1136/bmj.c3823

48. van Rossum CT, Shipley MJ, Hemingway H, et al. Seasonal variation in cause-specific mortality: Are there high-risk groups? 25-year follow-up of civil servants from the first Whitehall study. International Journal of Epidemiology 2001;30:1109–1116. doi:10.1093/ije/30.5.1109

49. Barnett AG, Dobson AJ, McElduff P. Cold periods and coronary events: an analysis of populations worldwide. J Epidemiol Community Health 2005;59:551–557. doi:10.1136/jech.2004.028514

50. Gerber Y, Jacobsen SJ, Killian JM. Seasonality and Daily Weather Conditions in Relation to Myocardial Infarction and Sudden Cardiac Death in Olmsted County, Minnesota, 1979 to 2002. J Am Coll Cardiol 2006;48:287–292. doi:10.1016/j.jacc.2006.02.065

51. Mackenbach JP. Health inequalities: Persistence and change in modern welfare states. 1st ed. Oxford University Press. 2019.

52. Hales S, Blakely T, Foster RH, et al. Seasonal patterns of mortality in relation to social factors. J Epidemiol Community Health 2012;66:379–384. doi:10.1136/jech.2010.111864

53. Turunen M, Iso-Markku K, Pekkonen M, et al. Statistical associations between housing quality and health among Finnish households with children - Results from two (repeated) national surveys. Sci Total Environ 2017;574:1580–1587. doi:10.1016/j.scitotenv.2016.08.194

54. Thompson WW, Shay DK, Weintraub E, et al. Mortality Associated With Influenza and Respiratory Syncytial Virus in the United States. JAMA 2003;289:179–186. doi:10.1001/jama.289.2.179

55. Warren-Gash C, Blackburn R, Whitaker H, et al. Laboratory-confirmed respiratory infections as triggers for acute myocardial infarction and stroke: a self-controlled case series analysis of national linked datasets from Scotland. Eur Respir J 2018;51:1701794. doi:10.1183/13993003.01794-2017

For peer review only - http://bmjopen.bmj.com/site/about/guidelines.xhtml

For peer review only

Figure 1. All-cause winter and nonwinter mortality rates, Finland 1971-2019, men and women aged 60 and over.

Figure 2. Rate differences (upper panel) and rate ratios (lower panel) of winter and nonwinter mortality rates with detected breakpoints (vertical dashed lines) and their confidence intervals, Finland 1971-2019, men and women aged 60 and over.

For peer review only

For peer review only - http://bmjopen.bmj.com/site/about/guidelines.xhtml

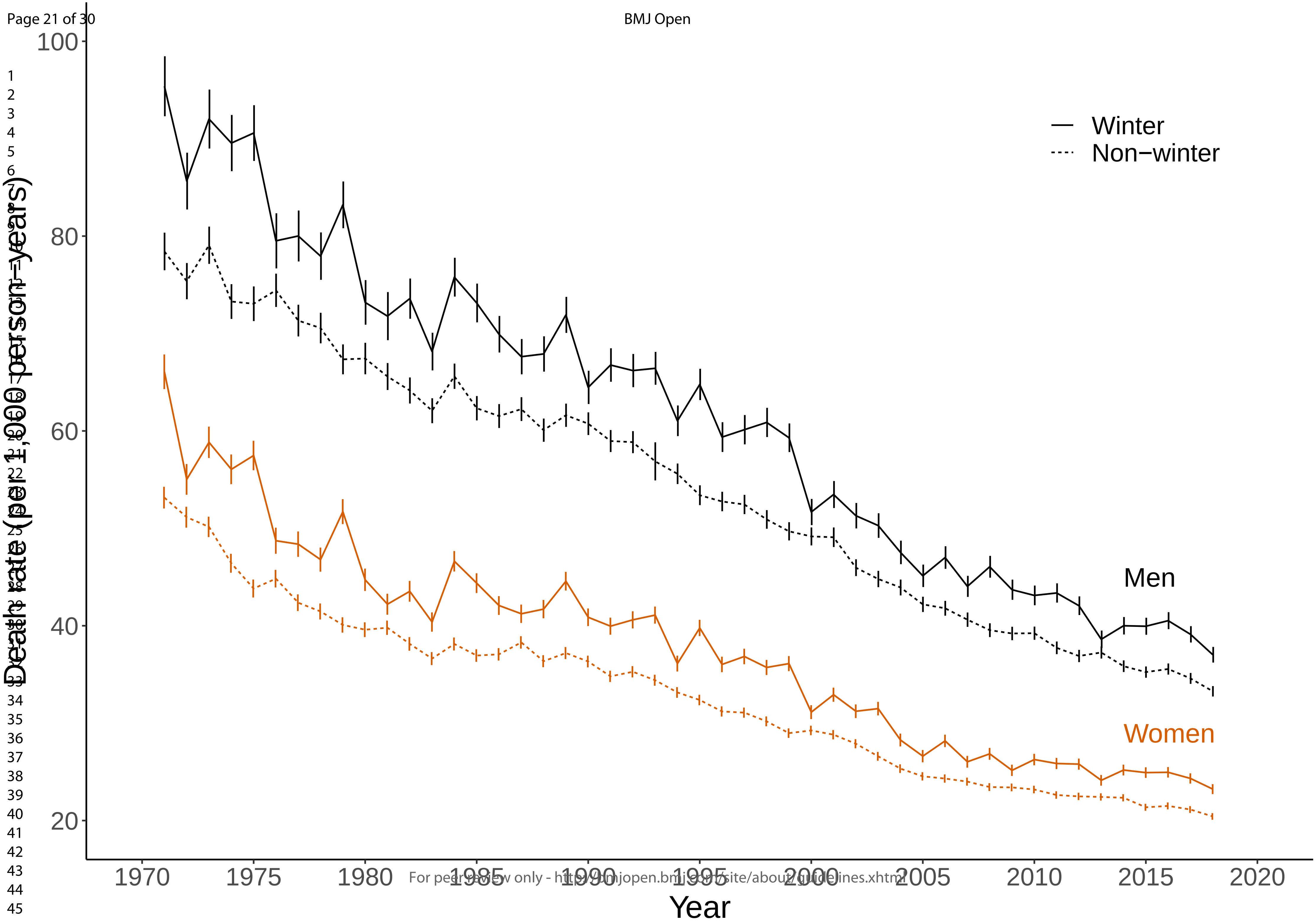
Death rate (per 1,000 person−years)
100
80
60
40
20
Year
1970
1975
1980
1985
1990
1995
2000
2005
2010
2015
2020
Winter
Non−winter
Men
Women

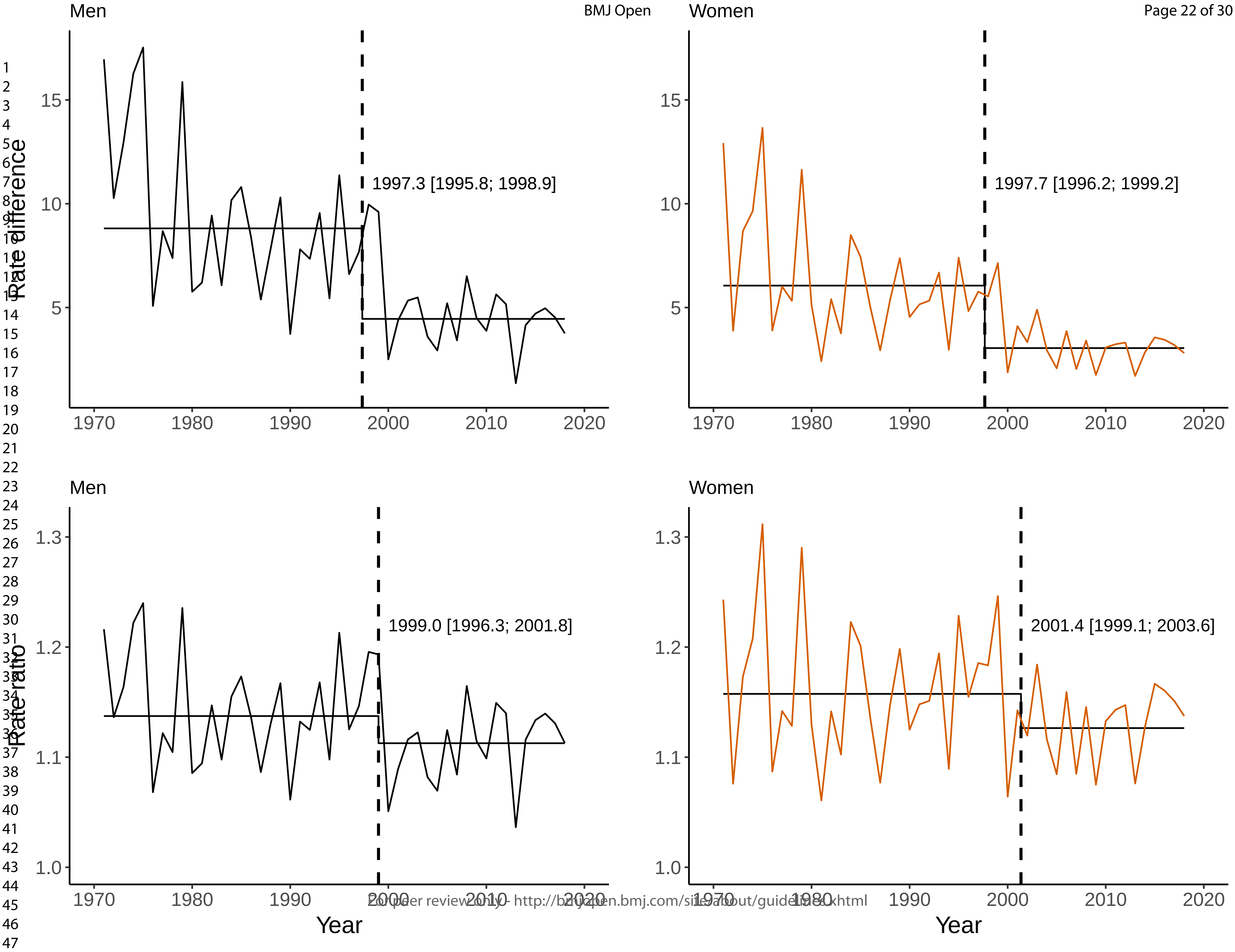
Men
Women
Rate difference
1997.3 [1995.8; 1998.9]
1997.7 [1996.2; 1999.2]
Rate ratio
1999.0 [1996.3; 2001.8]
2001.4 [1999.1; 2003.6]
Year
Year

SUPPLEMENTAL MATERIAL

Excess winter mortality in Finland 1971-2019 – a prospective register-based study on long-term trends and effect modification by sociodemographic characteristics and pre-existing health conditions

Ulla Suulamo, Hanna Remes, Lasse Tarkiainen, Mike Murphy, and Pekka Martikainen

Table S1. Categories of pre-existing health conditions.

Condition	Health care diagnosis, ICD-10 (Care register for health care HILMO, THL)	Refund categories, Finnish disease, or medicine codes (Register of Reimbursements for medicine purchases, Kela)
Dementia	F00-F03, F05.1, G30	307
Mental health disorders (excl. alcohol related)	F04-F99, excl. F10	Severe psychosis and other severe mental disorders (112)<br>Behavioural disorders in people with intellectual disabilities (113)
Diseases of the nervous system	G00-G29, G31-G99, excl. G31.2, G40.51, G62.1, G72.1	Parkinson's disease (110)<br>Epilepsy (111)<br>Multiple sclerosis (109)<br>Myasthenia gravis (108)<br>Neuralgia trigemini, neuralgia glossopharyngeal (119)<br>Narcolepsy (214)<br>Dimethyl fumarate, diroximel fumarate etc (157)<br>Retigabine and vigabatrin (181)<br>Brivaracetam, eslikarbazepin etc (182)<br>Lamotrigin and topiramate (183)<br>Rasagiline (193)<br>Rufinamide (199)
Diabetes	E10-E14	Diabetes (103)<br>Diabetes other than insulin therapy (215)
Diseases of the circulatory system (excl. alcohol related)	I00-I99, excl. I42.6	Chronic cardiac insufficiency (201)<br>Chronic coronary heart disease (206)<br>Chronic hypertension (205)<br>Chronic cardiac arrhythmias (207)
Diseases of the respiratory system	J00-J99, excl. J65	Chronic asthma and COPD (203)
Alcohol related diseases	F10, G31.2, G40.51, G62.1, G72.1, I42.6, K29.2, K70, K86.0, K85.2, O35.4, X45	
Accidents and violence (excl. alcohol related)	V01-Y89, excl. X45	

For peer review only - http://bmjopen.bmj.com/site/about/guidelines.xhtml

Table S2. Characteristics of the study population. Distribution according to status in 2000 and 2018, by age category.

	2000/01 (%)				2018/19 (%)			
	**60–69**	**70–79**	**80–89**	**90–**	**60–69**	**70–79**	**80–89**	**90–**
**Sociodemographic characteristics**								
Mean age (sd)	64.3 (2.9)	74.1 (2.8)	83.6 (2.8)	92.2 (2.3)	64.5 (2.9)	73.8 (2.9)	83.8 (2.8)	92.5 (2.5)
Sex								
Men	47.0	39.0	28.1	20.9	48.5	45.6	37.6	24.9
Women	53.0	61.0	71.9	79.1	51.5	54.4	62.4	75.1
Income quartile								
1 (lowest)	25.0	25.0	25.0	25.2	25.0	25.0	25.0	24.8
2	25.0	25.0	25.0	24.9	25.0	25.0	25.0	25.0
3	25.0	25.0	25.0	24.9	25.0	25.0	25.0	25.1
4 (highest)	25.0	25.0	25.0	24.9	25.0	25.0	25.0	25.0
Living arrangement								
Household of >1	74.6	60.7	39.3	25.5	70.2	65.3	46.4	23.5
Alone	24.5	37.0	51.2	47.0	28.2	32.8	47.2	57.5
Institution	1.0	2.3	9.5	27.4	1.5	1.9	6.4	19.1
Large regions								
Coast	37.1	35.0	36.8	40.8	38.9	39.6	36.6	36.8
South	28.0	29.7	30.4	29.7	27.0	28.1	28.9	30.6
Center	19.0	19.6	18.9	17.7	17.8	17.6	18.9	18.2
North	16.0	15.7	13.9	11.8	16.3	14.7	15.7	14.4
**Pre-existing health conditions**								
Dementia	0.2	1.5	4.4	7.2	0.4	2.5	12.0	22.4
Mental health disorders	3.7	3.8	3.9	2.9	4.0	3.6	3.8	3.5
Diseases of the nervous system	3.9	4.9	5.2	4.1	6.3	7.2	7.3	4.9
Diabetes	5.8	8.7	9.7	7.7	12.4	17.4	18.1	13.7
Circulatory	35.3	49.7	58.6	57.8	24.2	39.9	54.5	58.6
Respiratory	8.2	10.4	10.9	10.3	9.4	12.0	14.9	15.7
Alcohol related diseases	0.4	0.2	0.1	0.1	0.7	0.4	0.2	0.0
Accidents and violence	3.3	4.4	7.2	10.2	5.0	6.2	9.6	13.7
Other	24.7	32.4	38.8	36.3	29.3	39.4	47.4	45.6
None	42.5	29.3	21.1	21.7	43.4	28.7	16.6	13.4
Total	100.0	100.0	100.0	100.0	100.0	100.0	100.0	100.0
N	488,751	372,305	152,561	22,026	729,831	530,492	248,299	50,543

For peer review only - http://bmjopen.bmj.com/site/about/guidelines.xhtml

Table S3. Differences in winter mortality according to modifying factors (95% CI) by age category. Finland 2000-2019.

	**60–69** n=135,380	**70–79** n=224,573	**80–89** n=325,160	**90 and over** n=139,811
Winter-nonwinter rate ratio	1.06 (1.05, 1.07)	1.10 (1.09, 1.11)	1.14 (1.13, 1.15)	1.19 (1.17, 1.20)
**Sociodemographic characteristics**				
Sex				
Men	1.00	1.00	1.00	1.00
Women	1.01 (0.99, 1.03)	1.01 (0.99, 1.03)	**1.02 (1.00, 1.03)**	0.99 (0.97, 1.02)
Income quartile				
1 (lowest)	1.00	1.00	1.00	1.00
2	0.99 (0.96, 1.02)	1.00 (0.98, 1.03)	1.00 (0.98, 1.02)	1.01 (0.98, 1.04)
3	**0.96 (0.93, 0.99)**	1.01 (0.98, 1.03)	1.00 (0.98, 1.02)	1.00 (0.97, 1.03)
4 (highest)	0.98 (0.94, 1.01)	0.99 (0.96, 1.01)	1.00 (0.98, 1.02)	1.00 (0.97, 1.03)
Living arrangement				
Household of >1	1.00	1.00	1.00	1.00
Alone	1.01 (0.99, 1.03)	1.00 (0.98, 1.01)	1.01 (0.99, 1.02)	0.98 (0.96, 1.01)
Institution	**1.09 (1.04, 1.15)**	**1.07 (1.04, 1.10)**	**1.03 (1.01, 1.05)**	1.01 (0.98, 1.04)
Region				
Coast	1.00	1.00	1.00	1.00
South	1.02 (0.99, 1.05)	1.02 (1.00, 1.04)	1.01 (0.99, 1.03)	1.01 (0.98, 1.04)
Center	1.00 (0.97, 1.04)	1.02 (1.00, 1.05)	1.00 (0.98, 1.02)	1.00 (0.97, 1.03)
North	1.03 (0.99, 1.06)	1.02 (1.00, 1.05)	1.02 (1.00, 1.04)	1.01 (0.97, 1.04)
**Pre-existing health conditions**				
Dementia				
No	1.00	1.00	1.00	1.00
Yes	**1.07 (1.00, 1.15)**	**1.07 (1.04, 1.10)**	**1.04 (1.02, 1.05)**	1.01 (0.98, 1.03)
Mental health disorders				
No	1.00	1.00	1.00	1.00
Yes	**1.06 (1.02, 1.10)**	**1.04 (1.01, 1.07)**	1.02 (0.99, 1.05)	0.98 (0.93, 1.04)
Diseases of the nervous system				
No	1.00	1.00	1.00	1.00
Yes	**1.05 (1.01, 1.08)**	0.99 (0.97, 1.02)	0.99 (0.97, 1.02)	0.99 (0.94, 1.04)
Diabetes				
No	1.00	1.00	1.00	1.00
Yes	1.00 (0.97, 1.03)	1.02 (0.99, 1.04)	**1.02 (1.00, 1.04)**	1.00 (0.97, 1.04)
Circulatory				
No	1.00	1.00	1.00	1.00
Yes	0.99 (0.96, 1.01)	1.00 (0.99, 1.02)	1.00 (0.99, 1.02)	0.99 (0.97, 1.02)
Respiratory				
No	1.00	1.00	1.00	1.00
Yes	**1.03 (1.00, 1.06)**	**1.03 (1.01, 1.05)**	**1.02 (1.00, 1.04)**	1.01 (0.98, 1.04)
Alcohol related diseases				
No	1.00	1.00	1.00	1.00
Yes	0.98 (0.92, 1.04)	0.98 (0.90, 1.07)	0.87 (0.73, 1.05)	1.19 (0.68, 2.08)
Accidents and violence				
No	1.00	1.00	1.00	1.00
Yes	0.99 (0.95, 1.03)	0.99 (0.96, 1.02)	1.00 (0.98, 1.02)	1.02 (0.98, 1.05)
Other				
No	1.00	1.00	1.00	1.00
Yes	0.99 (0.97, 1.01)	**0.95 (0.93, 0.97)**	**0.98 (0.97, 1.00)**	**0.96 (0.94, 0.99)**
None				
No	1.00	1.00	1.00	1.00
Yes	1.01 (0.98, 1.04)	1.00 (0.98, 1.03)	0.99 (0.96, 1.01)	1.03 (1.00, 1.06)

Note: Models adjusted for winter, sex, age, income, living arrangement, region, year

For peer review only - http://bmjopen.bmj.com/site/about/guidelines.xhtml

For peer review only

The RECORD statement – checklist of items, extended from the STROBE statement, that should be reported in observational studies using routinely collected health data.

	Item No.	STROBE items	Location in manuscript where items are reported	RECORD items	Location in manuscript where items are reported
**Title and abstract**					
	1	(a) Indicate the study's design with a commonly used term in the title or the abstract (b) Provide in the abstract an informative and balanced summary of what was done and what was found	page 1-2	RECORD 1.1: The type of data used should be specified in the title or abstract. When possible, the name of the databases used should be included.<br><br>RECORD 1.2: If applicable, the geographic region and timeframe within which the study took place should be reported in the title or abstract.<br><br>RECORD 1.3: If linkage between databases was conducted for the study, this should be clearly stated in the title or abstract.	page 1-2
**Introduction**					
Background rationale	2	Explain the scientific background and rationale for the investigation being reported	page 3		
Objectives	3	State specific objectives, including any prespecified hypotheses	page 4		
**Methods**					
Study Design	4	Present key elements of study design early in the paper	page 4		
Setting	5	Describe the setting, locations, and relevant dates, including periods of recruitment, exposure, follow-up, and data collection	page 4		

For peer review only

Participants	6	*(a) Cohort study* - Give the eligibility criteria, and the sources and methods of selection of participants. Describe methods of follow-up<br>*Case-control study* - Give the eligibility criteria, and the sources and methods of case ascertainment and control selection. Give the rationale for the choice of cases and controls<br>*Cross-sectional study* - Give the eligibility criteria, and the sources and methods of selection of participants<br><br>*(b) Cohort study* - For matched studies, give matching criteria and number of exposed and unexposed<br>*Case-control study* - For matched studies, give matching criteria and the number of controls per case	page 4	RECORD 6.1: The methods of study population selection (such as codes or algorithms used to identify subjects) should be listed in detail. If this is not possible, an explanation should be provided.<br><br>RECORD 6.2: Any validation studies of the codes or algorithms used to select the population should be referenced. If validation was conducted for this study and not published elsewhere, detailed methods and results should be provided.<br><br>RECORD 6.3: If the study involved linkage of databases, consider use of a flow diagram or other graphical display to demonstrate the data linkage process, including the number of individuals with linked data at each stage.	page 4
Variables	7	Clearly define all outcomes, exposures, predictors, potential confounders, and effect modifiers. Give diagnostic criteria, if applicable.	page 4-5	RECORD 7.1: A complete list of codes and algorithms used to classify exposures, outcomes, confounders, and effect modifiers should be provided. If these cannot be reported, an explanation should be provided.	
Data sources/ measurement	8	For each variable of interest, give sources of data and details of methods of assessment (measurement).<br>Describe comparability of assessment methods if there is more than one group	page 4-5		

For peer review only

Bias	9	Describe any efforts to address potential sources of bias	page 13		
Study size	10	Explain how the study size was arrived at			
Quantitative variables	11	Explain how quantitative variables were handled in the analyses. If applicable, describe which groupings were chosen, and why	page 4-5		
Statistical methods	12	(a) Describe all statistical methods, including those used to control for confounding<br>(b) Describe any methods used to examine subgroups and interactions<br>(c) Explain how missing data were addressed<br>(d) *Cohort study* - If applicable, explain how loss to follow-up was addressed<br>*Case-control study* - If applicable, explain how matching of cases and controls was addressed<br>*Cross-sectional study* - If applicable, describe analytical methods taking account of sampling strategy<br>(e) Describe any sensitivity analyses	page 5-6		
Data access and cleaning methods		..		RECORD 12.1: Authors should describe the extent to which the investigators had access to the database population used to create the study population.	

For peer review only

				RECORD 12.2: Authors should provide information on the data cleaning methods used in the study.	
Linkage		..		RECORD 12.3: State whether the study included person-level, institutional-level, or other data linkage across two or more databases. The methods of linkage and methods of linkage quality evaluation should be provided.	page 5
**Results**					
Participants	13	(a) Report the numbers of individuals at each stage of the study (*e.g.*, numbers potentially eligible, examined for eligibility, confirmed eligible, included in the study, completing follow-up, and analysed)<br>(b) Give reasons for non-participation at each stage.<br>(c) Consider use of a flow diagram	page 7	RECORD 13.1: Describe in detail the selection of the persons included in the study (*i.e.,* study population selection) including filtering based on data quality, data availability and linkage. The selection of included persons can be described in the text and/or by means of the study flow diagram.	page 4-5
Descriptive data	14	(a) Give characteristics of study participants (*e.g.*, demographic, clinical, social) and information on exposures and potential confounders<br>(b) Indicate the number of participants with missing data for each variable of interest<br>(c) *Cohort study* - summarise follow-up time (*e.g.*, average and total amount)	page 7		
Outcome data	15	*Cohort study* - Report numbers of outcome events or summary measures over time<br>*Case-control study* - Report numbers in each exposure	page 9		

For peer review only

		category, or summary measures of exposure<br>*Cross-sectional study* - Report numbers of outcome events or summary measures			
Main results	16	(a) Give unadjusted estimates and, if applicable, confounder-adjusted estimates and their precision (e.g., 95% confidence interval). Make clear which confounders were adjusted for and why they were included<br>(b) Report category boundaries when continuous variables were categorized<br>(c) If relevant, consider translating estimates of relative risk into absolute risk for a meaningful time period	page 9		
Other analyses	17	Report other analyses done—e.g., analyses of subgroups and interactions, and sensitivity analyses	page 8		
**Discussion**					
Key results	18	Summarise key results with reference to study objectives	page 10		
Limitations	19	Discuss limitations of the study, taking into account sources of potential bias or imprecision. Discuss both direction and magnitude of any potential bias	page 13	RECORD 19.1: Discuss the implications of using data that were not created or collected to answer the specific research question(s). Include discussion of misclassification bias, unmeasured confounding, missing data, and changing eligibility over time, as they pertain to the study being reported.	page 13
Interpretation	20	Give a cautious overall interpretation of results considering objectives,	page 13		

For peer review only

		limitations, multiplicity of analyses, results from similar studies, and other relevant evidence			
Generalisability	21	Discuss the generalisability (external validity) of the study results	page 13		
**Other Information**					
Funding	22	Give the source of funding and the role of the funders for the present study and, if applicable, for the original study on which the present article is based	page 13		
Accessibility of protocol, raw data, and programming code		..		RECORD 22.1: Authors should provide information on how to access any supplemental information such as the study protocol, raw data, or programming code.	page 14

*Reference: Benchimol EI, Smeeth L, Guttmann A, Harron K, Moher D, Petersen I, Sørensen HT, von Elm E, Langan SM, the RECORD Working Committee. The REporting of studies Conducted using Observational Routinely-collected health Data (RECORD) Statement. *PLoS Medicine* 2015; in press.

*Checklist is protected under Creative Commons Attribution (CC BY) license.

For peer review only

Excess winter mortality in Finland in 1971-2019 – a register-based study on long-term trends and effect modification by sociodemographic characteristics and pre-existing health conditions

Journal:	*BMJ Open*
Manuscript ID	bmjopen-2023-079471.R1
Article Type:	Original research
Date Submitted by the Author:	05-Dec-2023

Complete List of Authors:	Suulamo, Ulla; University of Helsinki Faculty of Social Sciences, Helsinki Institute for Demography and Population Health<br>Remes, Hanna; University of Helsinki Faculty of Social Sciences, Helsinki Institute for Demography and Population Health<br>Tarkiainen , Lasse ; University of Helsinki Faculty of Social Sciences, Helsinki Institute for Demography and Population Health<br>Murphy, Michael; The London School of Economics and Political Science Department of Social Policy<br>Martikainen, Pekka; University of Helsinki Faculty of Social Sciences, Helsinki Institute for Demography and Population Health; Max-Planck-Institute for Demographic Research

**Primary Subject Heading**:	Epidemiology
Secondary Subject Heading:	Public health
Keywords:	Mortality, PUBLIC HEALTH, REGISTRIES, Risk Factors, Aged, EPIDEMIOLOGY

SCHOLARONE™ Manuscripts

I, the Submitting Author has the right to grant and does grant on behalf of all authors of the Work (as defined in the below author licence), an exclusive licence and/or a non-exclusive licence for contributions from authors who are: i) UK Crown employees; ii) where BMJ has agreed a CC-BY licence shall apply, and/or iii) in accordance with the terms applicable for US Federal Government officers or employees acting as part of their official duties; on a worldwide, perpetual, irrevocable, royalty-free basis to BMJ Publishing Group Ltd ("BMJ") its licensees and where the relevant Journal is co-owned by BMJ to the co-owners of the Journal, to publish the Work in this journal and any other BMJ products and to exploit all rights, as set out in our licence.

The Submitting Author accepts and understands that any supply made under these terms is made by BMJ to the Submitting Author unless you are acting as an employee on behalf of your employer or a postgraduate student of an affiliated institution which is paying any applicable article publishing charge ("APC") for Open Access articles. Where the Submitting Author wishes to make the Work available on an Open Access basis (and intends to pay the relevant APC), the terms of reuse of such Open Access shall be governed by a Creative Commons licence – details of these licences and which Creative Commons licence will apply to this Work are set out in our licence referred to above.

Other than as permitted in any relevant BMJ Author's Self Archiving Policies, I confirm this Work has not been accepted for publication elsewhere, is not being considered for publication elsewhere and does not duplicate material already published. I confirm all authors consent to publication of this Work and authorise the granting of this licence.

For peer review only

Original research

Excess winter mortality in Finland in 1971-2019 – a register-based study on long-term trends and effect modification by sociodemographic characteristics and pre-existing health conditions

Ulla Suulamo,[1,2] Hanna Remes,[1] Lasse Tarkiainen,[1] Mike Murphy,[3] and Pekka Martikainen[1,4,5]

Author Information

[1]Helsinki Institute for Demography and Population Health, Faculty of Social Sciences, University of Helsinki, Helsinki, Finland

[2]International Max Planck Research School for Population, Health and Data Science, Rostock, Germany

[3]Department of Social Policy, London School of Economics and Political Science, London, United Kingdom

[4]Max Planck Institute for Demographic Research, Rostock, Germany

[5]Max Planck – University of Helsinki Center for Social Inequalities in Population Health, Helsinki, Finland

Correspondence to: Ulla Suulamo, Helsinki Institute for Demography and Population Health, Faculty of Social Sciences, University of Helsinki, PO Box 42 (Unioninkatu 33), FIN-00014 Helsinki, Finland; ulla.suulamo@helsinki.fi

For peer review only

ABSTRACT

Objectives Excess winter mortality is a well-established phenomenon across the developed world. However, whether individual-level factors increase vulnerability to the effects of winter remains inadequately examined. Our aim was to assess long-term trends in excess winter mortality in Finland and estimate the modifying effect of sociodemographic and health characteristics on the risk of winter death.

Design Nationwide register study.

Setting Finland.

Participants Population aged 60 years and over resident in Finland in 1971-2019.

Outcome measures Age-adjusted winter and nonwinter death rates, and winter-to-nonwinter rate ratios and relative risks (multiplicative interaction effects between winter and modifying characteristics).

Results We found a decreasing trend in the relative winter excess mortality over five decades and a drop in the series around 2000. During 2000-19 winter mortality rates for men and women were 11% and 14% higher than expected based on nonwinter rates. The relative risk of winter death increased with age but did not vary by income. Compared with those living with at least one other person, individuals in institutions had a higher relative risk (1.07, 95% CI 1.05–1.08). Most pre-existing health conditions did not predict winter death, but persons with dementia emerged at greater relative risk (1.06, 95% CI 1.04–1.07).

Conclusions Although winter mortality seems to affect frail people more strongly – those of advanced age, living in institutions and with dementia – there is an increased risk even beyond the more vulnerable groups. Protection of high-risk groups should be complemented with population-level preventive measures.

Keywords: Excess Mortality; Trends; Effect Modification; Risk Factors

Strengths and limitations of this study

- We used high-quality, individual-level register data spanning nearly 50 years with minimal attrition and no recall bias.
- The use of register data allowed us to examine risk factors at the individual level.
- We used data on healthcare visits and reimbursement for medicine costs to examine whether those with pre-existing health conditions are particularly vulnerable.

For peer review only - http://bmjopen.bmj.com/site/about/guidelines.xhtml

- Using only the underlying cause of death may underestimate the total impact of respiratory infections and influenza-like illnesses on excess winter mortality.

INTRODUCTION

In temperate climates, mortality rates tend to be higher in winter compared to other times of the year. Although studies on long-term trends indicate that this winter excess has weakened over time [1-4], it remains a significant public health burden in many high-income countries, with winter increases in deaths of up to 28 per cent [5]. This burden – likely driven both by the direct effects of winter conditions on the most frail individuals, as well as increased seasonal pressure on the health care system and knock-on effects on other patients – may grow even greater in ageing populations, considering that the risk of winter death increases with age and is highest among the oldest-old [6, 7].

The increased risk of dying in winter is attributed to multiple factors, most importantly influenza-like communicable illnesses [8, 9] and low temperatures [10]. Consistent with these proximate risk factors, a winter peak in mortality is apparent for most major causes of death, but the largest seasonal fluctuations are seen for circulatory and respiratory diseases [11]. While such external effects drive the overall seasonal pattern, several non-seasonal risk factors have been found to importantly modify an individual's vulnerability to the effects of winter. Previous evidence is clear in relation to age: excess winter mortality especially affects older people, gradually increasing with age [12]. Besides age, factors such as low income, inadequate housing conditions and fuel poverty [13] have been associated with increased adverse health outcomes in winter, especially in countries with thermally inefficient housing stocks such as Portugal [14] and the UK [15]. Although based on limited evidence, living arrangements are another potentially important factor in differential mortality, and some previous findings suggest a higher risk of excess winter mortality for those living alone [12] or in institutions [16-18].

The importance of non-seasonal risk factors is further demonstrated by cross-country comparisons, which suggest that absolute winter temperatures matter little, as countries with milder winters generally experience much greater wintertime excess than countries with colder climates [5, 19]. These somewhat counterintuitive findings have led investigators to suggest that populations' ability to protect themselves is what ultimately determines the extent of winter mortality [20], and indicate that winter excess deaths are, to an important degree, preventable [5, 7, 21].

Despite the avoidable character of a substantial fraction of excess winter mortality, uncertainty still exists whether the winter excess mainly affects frail individuals who are at greater risk of dying soon in any case. Those with pre-existing health conditions are often considered particularly vulnerable, but empirical evidence to confirm this remains scarce. One previous study [22] assessing the determinants of vulnerability to excess winter mortality found evidence for an

For peer review only - http://bmjopen.bmj.com/site/about/guidelines.xhtml

For peer review only

increased risk of winter death only for those with a history of respiratory illness but not for those with other health conditions. Addressing whether excess winter mortality mainly affects the frail remains thus an important area of further inquiry with potentially important policy implications: identifying those most affected may help evaluate how preventable these excess deaths are and guide the development of targeted prevention measures.

In international comparison, northern European countries with relatively cold winters show lower levels of excess winter mortality which has been interpreted to arise from a combination of socioeconomic, housing, and behavioural factors [19]. Despite lower levels of excess, the winter period is nevertheless acknowledged to pose a range of health challenges [23], and population ageing makes it likely that these challenges will become more pressing. Little is, however, known about how excess winter mortality differs by individual-level social and health characteristics in a societal context with high-quality housing at all income levels and universal access to health care services. Using individual-level data on the total population, we examined long-term trends in excess winter mortality in Finland at ages 60 and above in 1971–2019 and assessed differences in the winter excess by cause of death and the modifying effect of sociodemographic characteristics and pre-existing health conditions in 2000–2019.

METHODS

Data

The study was based on individual-level data for the total population of Finland from August 1971 to July 2019. This data were drawn from censuses and population registers that Statistics Finland had linked to cause-of-death records and health care registers using personal identification numbers. For 1971-1987 sociodemographic information was obtained from the end-of-year census records (1970/1975/1980/1985) and from the year 1988 onwards from the end-of-year population records. We restricted the analysis to individuals aged 60 years and over at the end of each year from 1971 to2018.

We defined winter as December-March following previous studies in the northern hemisphere [13]. These months also meet the definition of thermal winter in Finland with daily mean temperatures below 0°C (32°F). On average, in central and southern Finland, where most of the population resides, thermal winter begins around late November and ends in late March. Nonwinter months were defined as the four months on each side of this winter period (August-November, April-July). Following this definition, our study period to analyse trends over time ran from August 1971 to July 2019, 48 years in total. For each year, we followed mortality from the beginning of August to end of July next year.

For peer review only - http://bmjopen.bmj.com/site/about/guidelines.xhtml

For the more recent 19-year period August 2000 – July 2019, we assessed whether and how excess winter mortality was modified by sex, age, living arrangement, income, region, and pre-existing health conditions. Age was categorized into groups 60-69, 70-79, 80-89 and 90 years and over. Living arrangement distinguished between those living in households with at least one other person, alone or in institutions. Household disposable income was categorized into quartiles in the population aged 60 and over and adjusted for the household composition [24]. For persons living in institutions, we used personal disposable income. Information on region of residence (n=19) was aggregated into four larger regions (coastal, southern, central, and northern) according to average winter temperatures. Living arrangement, income and region were measured at the end of the preceding calendar year, whereas age corresponded to attained age in July of each year.

We used 11 dichotomous indicators of pre-existing health conditions: dementia, mental disorders, cancer, diseases of the nervous system, diabetes, diseases of the circulatory system, diseases of the respiratory system, alcohol-related causes, injuries due to accidents and violence, other health conditions and an overall measure of any health condition. These conditions were identified from two sources covering the years 1999-2017: the health care register of the Finnish Institute for Health and Welfare and the medication reimbursement register of the Social Insurance Institution of Finland. We included data on inpatient and specialized outpatient care episodes that occurred during the preceding calendar year (at least seven months before the start of mortality follow-up) and for which any of the aforementioned conditions were recorded as the primary discharge diagnoses. The health conditions were classified based on the International Classification of Diseases (ICD) 10th Revision. Information on the right to special reimbursement for medicine costs due to diagnosed chronic medical conditions during the preceding calendar year was collected from the medication reimbursement register of the Social Insurance Institution of Finland following their disease and medication classification. For exact coding of care episodes and reimbursement categories see Table S1 in the Supplemental Material. A pre-existing health condition was considered present if there was a record in either of the two registers and each individual could contribute to several categories.

In addition to all-cause mortality, we assessed winter excess mortality separately for deaths due to diseases of the circulatory system (ICD 10 codes I00-I425, I427-I99), diseases of the respiratory system (J00-J64, J66-J99), dementia (F01, F03, G30, R54), other internal causes, and external causes (V01-X44, X46-Y89). The coding of causes of death was based on Statistics Finland's cause of death classification, which is harmonized across different revisions of the ICD.

Patient and Public Involvement

All data was collected for routine administrative purposes and, therefore, public involvement was not feasible.

For peer review only - http://bmjopen.bmj.com/site/about/guidelines.xhtml

Statistical analysis

To obtain person-days at risk for each winter and nonwinter period, we created an expanded dataset by splitting each year on April 1st and December 1st. Deaths were allocated to the appropriate winter and nonwinter periods using exact dates of death.

To assess trends over time, we collapsed the expanded dataset by summing the death and person-time counts over sex, age, year, and winter and nonwinter periods. We then calculated and plotted age-adjusted annual winter and nonwinter death rates with 95% confidence intervals (CI) in 1971-2019 using the age structure of the entire period as the standard population. Both absolute and relative differences between winter and nonwinter rates were computed. To identify shifts in the trends, we explored the presence and location of breakpoints in the series of absolute and relative differences. We applied segmented linear regression analysis with one break on the cumulative values using the "segmented" package [25] in R software version 4.2.2 (R Core Team).

To study the extent to which individual characteristics modified the risk of excess winter mortality, we focused on the more recent period from August 2000 to July 2019. We assessed effect modification using Poisson regression with interactions between the winter indicator and the covariates. These interaction effects, interpreted as relative risks, show how the effect of winter varies in multiplicative terms between different categories of modifying factors and the baseline reference group. Each potential effect modifier was studied separately, adjusting the models for all the other covariates. To address potential issues of overdispersion in the Poisson regression models, robust, clustered standard errors were employed, with each cluster defined as an individual.

RESULTS

Over the study period from August 1971 through July 2019, 1,902,824 deaths were recorded, of which 36% occurred during winter.

The age-adjusted winter death rate fell from 95 deaths per 1,000 person-years among men and 66 among women in 1971 to 37 and 23 in 2018, respectively (Figure 1). The nonwinter death rate was consistently below that of winter throughout the observation period and decreased from 78 to 33 among men and from 53 to 20 among women.

Absolute and relative differences between winter and nonwinter mortality (Figure 2) showed considerable year-to-year fluctuations. Overall, mortality declined over time, as did the absolute difference between winter and nonwinter mortality rates - from an average of around 15 deaths per 1,000 person-years to below five. Relative differences fluctuated between a winter excess of 31% (women year 1975) and only 4% (men year 2013), showing a clear downward trend. Changes in absolute differences for women were somewhat smaller than for men, and vice versa for relative differences, but peaks and troughs occurred in the same years for both sexes.

For peer review only - http://bmjopen.bmj.com/site/about/guidelines.xhtml

The values of both absolute and relative differences oscillated at lower levels towards the end of the study period, and the year-to-year variation became less pronounced. A breakpoint analysis indicated a change in the series around the year 2000 for both absolute and relative differences. The drop in the mean rate difference after the estimated break was nearly 50%, whereas for rate ratios, the drop was smaller.

Descriptive information about the study population at the end of the more recent period 2000-2019 shows population ageing and an increasing share of men (Table 1). Most health conditions have become more common, except diseases of the circulatory system. The percentage of individuals recorded with dementia increased from 1.4 to 3.7%, reflecting the ageing population. However, the proportion with any recorded health condition during the previous calendar year remained at two thirds. Living arrangements were stable, with around 3% living in institutions, one-third alone, and the rest with at least one other person.

Table 1. Changes in sociodemographic characteristics and pre-existing health conditions between 2000/01 and 2018/19, population aged 60 and over.

	2000/01 (%)	2018/19 (%)
**Sociodemographic characteristics**		
Mean age (sd)	71.2 (8.1)	71.6 (8.4)
Sex		
Men	40.8	45.0
Women	59.2	55.0
Age category		
60–69	47.2	46.8
70–79	35.9	34.0
80–89	14.7	15.9
90 and over	2.1	3.2
Income quartile		
1 (lowest)	25.0	25.0
2	25.0	25.0
3	25.0	25.0
4 (highest)	25.0	25.0
Living arrangement		
Household of >1	63.3	63.2
Alone	33.4	33.8
Institution	3.3	3.0
Large regions		
Coast	36.4	38.7
South	29.0	27.8
Center	19.2	17.9
North	15.5	15.6
**Pre-existing health conditions**		
Dementia	1.4	3.7
Mental health disorders	3.7	3.8
Cancer	4.3	7.5
Diseases of the nervous system	4.4	6.7
Diabetes	7.4	15.0
Diseases of the circulatory system	44.4	35.5
Diseases of the respiratory system	9.4	11.4

For peer review only - http://bmjopen.bmj.com/site/about/guidelines.xhtml

For peer review only

Alcohol related causes	0.3	0.5
Accidents and violence	4.4	6.4
Other than specified above	27.8	33.4
Any health condition	65.9	67.2
Total	100.0	100.0
N	1,035,643	1,559,165

In 2000-2019 all-cause mortality during winter occurred at an age- and sex-adjusted rate of 49.8 per 1,000 person-years among men, and 31.4 per 1,000 person-years among women, which was 11% and 14% higher than in the nonwinter period, respectively (Table 2). The winter excess varied by cause of death. It was observed for all cause of death groups, except for external causes among men, and was strongest for respiratory disease deaths. For both men and women, more deaths due to a respiratory cause occurred during the winter period (26% and 40% excess, respectively) than expected based on the nonwinter average. Those who died from dementia showed the second highest winter excess (men 21%, women 22%), exceeding that of circulatory disease deaths (men 13%, women 14%).

Table 2. Age- and sex-adjusted death rates in winter and nonwinter periods and rate ratios, by cause of death and sex. Finland 2000-2019, ages 60 and over.

	Men				Women			
	Deaths	Rate per 1,000 person years		Winter-nonwinter rate ratio	Deaths	Rate per 1,000 person years		Winter-nonwinter rate ratio
		Winter	Nonwinter			Winter	Nonwinter	
Circulatory	160,087	21.7	19.3	1.13 (1.12, 1.14)	190,933	13.5	11.8	1.14 (1.13, 1.15)
Respiratory	24,943	3.5	2.8	1.26 (1.23, 1.30)	18,236	1.5	1.1	1.40 (1.35, 1.44)
Dementia	36,659	6.0	4.9	1.21 (1.18, 1.24)	82,108	5.7	4.7	1.22 (1.20, 1.24)
Other internal	132,285	14.9	14.3	1.04 (1.03, 1.06)	131,892	9.4	8.9	1.06 (1.05, 1.07)
External	30,191	3.3	3.2	1.01 (0.99, 1.04)	17,590	1.3	1.2	1.06 (1.03, 1.10)
All causes	384,165	49.5	44.8	1.11 (1.10, 1.11)	440,759	31.4	27.6	1.14 (1.13, 1.14)

Table 3 shows age-adjusted winter and nonwinter death rates and the winter/nonwinter rate ratios for different categories of potential effect modifiers, adjusted for the other sociodemographic characteristics. For the winter/nonwinter rate ratios, we also show the multiplicative interaction effects for each covariate, i.e., the effect of winter relative to the ratio of the baseline reference group. The adjusted risk of dying in winter was slightly higher for women (1.14) than men (1.11), translating to a relative excess winter mortality that was 1.03 times higher among women than men (relative risk RR 1.03, 95% CI 1.02–1.04). The differences were clear between age groups with the most notable winter effects among the oldest-old, but there was no evidence that the risk of excess winter mortality varied by income and only weak evidence of differences between regions. The risk of dying in winter was relatively higher for persons living in institutions (RR 1.07, 95% CI 1.05–

For peer review only - http://bmjopen.bmj.com/site/about/guidelines.xhtml

1.08), but those who lived alone did not differ much from those living with at least one other person. Overall, simultaneous adjustment for all covariates had very minor effects on the results (results from unadjusted models not shown).

All analysed categories of pre-existing health conditions were associated with higher risk of death during both winter and nonwinter periods. However, the multiplicative interaction effects of winter remained modest for nearly all health conditions. The most substantial excess mortality in winter was found for persons with a history of dementia with an adjusted winter/nonwinter rate ratio of 1.17 (95% CI 1.16–1.19) and a winter effect that was 1.06 times higher than among those without dementia (RR 1.06 95% CI 1.04–1.07). Other categories of health conditions that seemed to slightly increase the risk of dying in winter included diseases of the respiratory system (RR 1.02, 95% CI 1.02-1.03) and mental disorders (RR 1.02, 95% CI 1.00-1.04). For alcohol-related causes and those with cancer, the effect was the other way around with relative risks of 0.93 (95% CI 0.89–0.98) and 0.93 (95% CI 0.92, 0.94), respectively.

Additional age-category-specific results (Tables S2 and S3) showed that while excess winter mortality increased with age, indications of effect modification by the analysed sociodemographic and health characteristics were mostly similar. Differences in the effect of winter were, however, more apparent among the younger age categories, whereas among 90-year-olds, no effect modification was observed.

Table 3. Death rates in winter and nonwinter periods, rate ratios, and relative risks of excess winter mortality by individual characteristics. Finland 2000-2019, men and women aged 60 and over.

	Age adjusted rate per 1000 person years		Full adjusted winter-nonwinter rate ratio (95% CI)[a]	Full adjusted relative risk (RR) (95% CI)[a,b]
	Winter (n=295,236)	Nonwinter (n=529,688)		
**Sociodemographic characteristics**				
All	37.8	33.7	1.12 (1.12, 1.13)	
Sex				
Men	49.5	44.8	1.11 (1.10, 1.11)	1.00
Women	31.4	27.6	1.14 (1.13, 1.14)	**1.03 (1.02, 1.04)**
Age category				
60–69	11.9	11.2	1.06 (1.05, 1.07)	1.00
70–79	30.7	27.9	1.10 (1.09, 1.11)	**1.04 (1.02, 1.05)**
80–89	94.6	83.2	1.14 (1.13, 1.15)	**1.07 (1.06, 1.09)**
90 and over	269.8	228.0	1.19 (1.17, 1.20)	**1.12 (1.10, 1.13)**
Income quartile				
1 (lowest)	41.5	37.2	1.12 (1.11, 1.13)	1.00
2	38.5	34.3	1.12 (1.11, 1.13)	1.00 (0.99, 1.02)
3	37.9	33.7	1.12 (1.11, 1.13)	1.01 (0.99, 1.02)
4 (highest)	32.9	29.2	1.13 (1.11, 1.14)	1.01 (0.99, 1.02)
Living arrangement				
Household of >1	31.0	28.0	1.10 (1.10, 1.11)	1.00
Alone	34.3	30.6	1.12 (1.11, 1.13)	**1.01 (1.00, 1.02)**
Institution	110.1	93.6	1.18 (1.16, 1.19)	**1.07 (1.05, 1.08)**

For peer review only - http://bmjopen.bmj.com/site/about/guidelines.xhtml

Region				
Coast	36.8	33.1	1.11 (1.10, 1.12)	1.00
South	37.5	33.2	1.13 (1.12, 1.14)	**1.02 (1.00, 1.03)**
Center	38.9	34.8	1.12 (1.11, 1.13)	1.01 (0.99, 1.02)
North	39.3	34.7	1.13 (1.12, 1.15)	**1.02 (1.01, 1.03)**
**Pre-existing health conditions**				
Dementia				
No	34.0	30.6	1.11 (1.11, 1.12)	1.00
Yes	78.9	67.2	1.17 (1.16, 1.19)	**1.06 (1.04, 1.07)**
Mental health disorders				
No	36.5	32.6	1.12 (1.12, 1.13)	1.00
Yes	69.6	60.9	1.14 (1.12, 1.16)	**1.02 (1.00, 1.04)**
Cancer				
No	34.9	30.7	1.14 (1.13, 1.14)	1.00
Yes	74.1	70.4	1.05 (1.04, 1.07)	**0.93 (0.92, 0.94)**
Diseases of the nervous system				
No	36.1	32.2	1.12 (1.12, 1.13)	1.00
Yes	65.6	58.7	1.12 (1.10, 1.13)	0.99 (0.98, 1.01)
Diabetes				
No	35.2	31.4	1.12 (1.12, 1.13)	1.00
Yes	57.0	50.5	1.13 (1.12, 1.14)	1.01 (1.00, 1.02)
Circulatory				
No	29.9	26.8	1.12 (1.11, 1.13)	1.00
Yes	45.5	40.4	1.13 (1.12, 1.13)	1.01 (1.00, 1.02)
Respiratory				
No	34.7	31.0	1.12 (1.11, 1.12)	1.00
Yes	61.2	53.6	1.14 (1.13, 1.15)	**1.02 (1.01, 1.03)**
Alcohol related				
No	37.5	33.4	1.12 (1.12, 1.13)	1.00
Yes	169.7	161.8	1.05 (1.00, 1.10)	**0.93 (0.89, 0.98)**
Accidents and violence				
No	36.4	32.5	1.12 (1.12, 1.13)	1.00
Yes	56.0	49.7	1.13 (1.11, 1.14)	1.00 (0.99, 1.02)
Other than specified above				
No	34.1	30.1	1.13 (1.13, 1.14)	1.00
Yes	44.2	39.9	1.11 (1.10, 1.12)	**0.98 (0.97, 0.99)**
Any health condition				
No	19.2	17.2	1.12 (1.11, 1.13)	1.00
Yes	43.7	38.9	1.12 (1.12, 1.13)	1.00 (0.99, 1.02)

[a]Models are adjusted for sex, age, income, living arrangement, region, and year.

[b]Relative risks in the last column are the interaction terms of the winter indicator and the effect modifier. These show the relative differences in the effect of winter on mortality between effect modifier categories.

DISCUSSION

Main findings

For peer review only - http://bmjopen.bmj.com/site/about/guidelines.xhtml

With individual-level total population data covering nearly half a century, we examined long-term trends in excess winter mortality in Finland and assessed how individual-level characteristics may modify the risk of dying in winter. We showed that both winter and nonwinter death rates steadily declined in 1971-2019, but winter rates remained above nonwinter rates through the period. All-cause mortality was 11% and 14% higher during winter compared to nonwinter months in 2000-19 for men and women, respectively, with highest excess in respiratory and dementia related causes of death. We found modest effect modification by sociodemographic characteristics and pre-existing health conditions. Advanced age, living in institutions, and dementia increased the risk of winter mortality, while income level and most health conditions made no difference.

Interpretation of the results

Previous research has documented downward trends in excess winter mortality during most of the 20th century [2, 3, 26, 27]. The declining trend has been attributed to general socioeconomic progress, improved housing conditions and healthcare systems. According to previous research, differences between winter and summer mortality started to decline relatively early in Finland, in the 1930s [28], and a slowly decreasing trend was also reported during the latter part of the century, until 1995 [29]. Although not directly comparable to ours because of different definition of the winter excess (deaths in September-March compared to the month of lowest mortality) and age ranges considered, these previous results do align with our findings. In addition, with our results extending over 20 years further, we report a clear attenuation in the year-to-year variability during the last two decades and a downturn in the rate ratios around the year 2000.

Influenza-like illnesses are an important contributor to excess winter mortality, some studies showing that it is especially these – and not cold severity – that nowadays explain the year-to-year variations in the winter excess [30, 31]. There are at least two influenza-related factors that may have contributed to the smaller winter mortality fluctuations observed towards the end of the study period. During the first decade of the 21st century, seasonal influenza activity and related mortality were relatively low compared to previous decades [32]. Also, since 2002, influenza vaccines have been administered as part of the national vaccination programme in Finland for all those aged 65 years and older. Vaccination is considered the best available intervention to prevent influenza illness, and some previous studies have reported that the influenza vaccine significantly reduces mortality [33, 34]. Although more research is needed as the mortality benefits of influenza vaccine, especially among the older population, have been questioned [35, 36], our results are consistent with the idea that both lower influenza activity of recent decades and introduction of vaccinations may have reduced excess winter mortality peaks.

Age is known to play an important role for excess winter mortality [12, 37], and of all the individual-level effect modifiers included in our analysis, older age showed the strongest and clearest influence. The increasing risk with age is commonly interpreted as a reflection of increasing frailty and vulnerability towards adverse environmental stresses as age advances [38].

For peer review only - http://bmjopen.bmj.com/site/about/guidelines.xhtml

Accordingly, we found that persons living in institutions were likelier to die in winter. As previous studies with similar findings from France [16], Netherlands [17] and England and Wales [18] note, those living in institutions are rarely exposed to outside weather and cold. However, they are likely to spend more time indoors within confined environments, which may increase the spread of infections [39]. Furthermore, the prevalence of frailty is considerably high among older adults living in residential care [40], indicating their increased risk of mortality and vulnerability to external stressors [41].

Apart from frailty associated with advanced age, it is generally assumed that excess winter mortality most importantly affects persons with pre-existing health conditions. Nevertheless, previous literature approaching the topic by including information about the prior health of those dying in winter is scarce. In general, our findings showed very weak or no evidence for increased risk for dying in winter among those with pre-existing conditions. Only persons with dementia emerged as being at greater risk, a finding in line with few previous studies. Our results concerning dementia mortality also support these findings, as those who died from dementia showed the second-highest winter excess of around 21%. Previously, Liddell and co-authors [42] have also reported almost a third more dementia deaths in winter compared to nonwinter times in the UK. While a tendency to attribute deaths to the underlying chronic condition instead of a respiratory infection could explain some of the excess, another possible interpretation is that those with dementia have difficulties in communicating changes in their health status to relatives and care staff. In a study on susceptibility to cold, Zanobetti and co-authors [43] found that in the US, persons with previous admission for dementia were at greater risk of dying on extremely cold days and it has been speculated that this may relate to reduced perception of and protection from cold [44]. Living in institutions, as discussed above, has also been suggested to play a role. A recent study [45] on COVID-19 pandemic-related excess mortality in the UK proposed that the highest relative increases of death for individuals with dementia may be attributed to higher exposure to seasonal respiratory viruses due to their more frequent residency in institutional settings. However, supplementary analyses (Table S4) in this study showed that effect modification of winter mortality by age or pre-existing diagnosis of dementia was in fact modest or non-existent among those living in institutions. These findings suggest that the elevated excess winter mortality in individuals with pre-existing dementia cannot be solely attributed to institutional living.

The risk of dying in winter was not significantly increased for those with other pre-existing health conditions. Not even those with respiratory or circulatory conditions seemed significantly more vulnerable, although persons with these conditions are traditionally assumed to face a particularly high risk of death in winter. This common assumption may partly arise from considering stated causes of death on the death certificate as evidence of pre-existing conditions and thus interpreting the findings that show high excess winter mortality for cardiovascular and respiratory causes as an indication that individuals with pre-existing cardiovascular or respiratory disease are more susceptible to the adverse effects of winter. We also found the highest excess winter mortality in

For peer review only - http://bmjopen.bmj.com/site/about/guidelines.xhtml

deaths due to diseases of the respiratory system and a somewhat lesser excess risk in deaths due to circulatory diseases, but a similar excess risk did not apply to people diagnosed with these conditions in the preceding year. Previous evidence on the effect of pre-existing health conditions – primarily based on studies on cold-related mortality and not on excess winter mortality specifically – is conflicting. For example, while Wilkinson and co-authors found that pre-existing respiratory disease increased the risk of a winter death, and Schwartz [46] reported elevated risks on cold days for persons with chronic obstructive pulmonary disease, other empirical studies have found no higher risks than average [47] and even lower than average risks have been reported [43]. Similarly, for cardiovascular conditions, there is some evidence both for [48, 49] and against [46, 50, 51] an increased risk of winter death. One possible explanation for not detecting higher risks for most of the pre-existing health conditions may be that persons with a diagnosis and previous healthcare contacts are the ones who receive most benefit from preventive measures. If true, this suggests potential for prevention among the apparently healthy population.

While there is a well-known link between socioeconomic position and overall mortality [52], we found no evidence to suggest a socioeconomic gradient in vulnerability to excess winter mortality when measured by income. Instead, the increase in mortality in winter was similar in all income categories. Previous studies regarding the association between socioeconomic factors and excess winter mortality have provided conflicting results [13]. Hales and co-authors [53], for example, found that the risk of winter death in New Zealand was higher for those in the lowest income tertile compared to those in the highest tertile. Many of the proposed mechanisms through which lower socioeconomic position and income may increase the risk of winter death are linked to housing conditions and factors such as central heating and fuel poverty [13]. However, it has also been noted that low socio-economic position does not always go hand in hand with the poorest quality housing, as in some countries, e.g. the UK, social housing may even have better than average energy efficiency characteristics [22]. In Finland, housing is generally of high quality, and conditions are relatively good at all income levels. Homes and buildings are well insulated, and most households can keep their homes adequately warm regardless of low outside temperatures [54], which probably partly explains why no income gradient was observed in our study. Similar conclusions were drawn from a previous study in another Nordic country, Denmark [12]. Future studies should, however, assess possible interactions between income and living arrangements in diverse social settings to gain a more comprehensive understanding of their effects on excess winter mortality.

Methodological considerations

Our study is based on full-coverage high-quality, population-level register data with minimal attrition and no underreporting or recall bias. The use of register data allowed us to examine risk factors at the individual instead of aggregate level, overcoming a key limitation of most previous work on excess winter mortality. Nevertheless, we recognize that identifying persons with pre-existing health conditions based on healthcare visits in specialized care and reimbursement for

For peer review only - http://bmjopen.bmj.com/site/about/guidelines.xhtml

medicine costs may present some limitations as these records may partly reflect differences in health-seeking behaviour and ignore less severe health problems. Another potential cause of concern relates to the timing of the measurement. If we measured health too close to death, we might be capturing the beneficial effect of being monitored by healthcare professionals or the results could be affected by a survivor bias. Our main results were based on measuring health conditions during the preceding calendar year (a 7-month "quarantine" period from January-July). We replicated the analysis measuring health closer (no quarantine period before start of follow up) and further back (quarantine period extended to two years). Results, however, remained unchanged, indicating that the timing of the measurement is not driving the small effects.

Our analyses on winter excess mortality in different causes of death were based solely on the underlying cause of death. Thus our findings may significantly underestimate the total impact of respiratory infections and influenza-like illnesses on excess winter mortality [21]. Deaths are rarely coded as influenza in death certificates [55] but instead attributed to chronic diseases, especially among the older population. Nevertheless, respiratory infections are important triggers of, for instance, cardiovascular problems [56]. To better understand the mechanisms behind excess winter mortality, future research should consider information on contributory causes of death to assess the indirect triggering effect of respiratory diseases.

Conclusions and implications

In conclusion, with high-quality individual-level total population data, we showed that winter mortality has declined since 1971, and particularly after year 2000, but the winter mortality rates have remained above nonwinter rates throughout the past five decades. We provide robust evidence that those with advanced age, living in institutions, and dementia had a particularly high risk of winter mortality. The subgroup differences were relatively small overall, suggesting an increased risk of winter death even beyond the more vulnerable subgroups. In the context of population ageing and increasing numbers of individuals surviving into ages where excess winter mortality reaches high levels, public health action to protect high-risk groups may have major population-level benefits.

Contributors US, HR, LT, MM and PM contributed to the conception and design of the study. PM acquired the data. US conducted the literature review, prepared the data, conducted the analyses, and wrote the first version of the manuscript. All authors critically revised, commented, and edited the draft versions of the manuscript for important intellectual content. All authors have approved the final version to be published and agree to be accountable for all aspects of the work. US is responsible for the overall content as guarantor.

Funding US was supported by Jenny and Antti Wihuri Foundation (grant numbers 00210374 and 0022038). PM was supported by the European Research Council under the European Union's

For peer review only - http://bmjopen.bmj.com/site/about/guidelines.xhtml

Horizon 2020 research and innovation programme (grant agreement number 101019329), the Strategic Research Council (SRC) within the Academy of Finland grants for ACElife (352543-352572) and LIFECON (308247), and grants to the Max Planck – University of Helsinki Center from the Jane and Aatos Erkko Foundation (210046), the Max Planck Society (5714240218), University of Helsinki (77204227), and Cities of Helsinki, Vantaa and Espoo.

Disclaimer The study does not necessarily reflect the Commission's views and in no way anticipates the Commission's future policy in this area. The funders had no role in the study design, data collection and analysis, decision to publish, or preparation of the manuscript.

Competing interests None declared

Patient consent Not required.

Ethics approval The use of the anonymized register data has been approved by the ethics boards of Statistics Finland and FinData (permissions TK-53-1490-18 and THL/2180/14.02.00/2020). The register data are originally collected for administrative and statistical purposes. No informed consent is required for register-based studies in Finland.

Data sharing Data are not publicly available. Due to data protection regulations of the register-holders, the authors are unable to make the data available to third parties. In order to use the data, researchers and research institutions need to apply for a license that authorizes its use for research purposes. Access to the data can be requested by contacting the register holders.

Acknowledgements US gratefully acknowledges the resources provided by the International Max Planck Research School for Population, Health and Data Science (IMPRS-PHDS).

REFERENCES

1. Almendra R, Perelman J, Vasconcelos J, et al. Excess winter mortality and morbidity before, during, and after the Great Recession: the Portuguese case. Int J Biometeorol 2019;63:873–883. doi:10.1007/s00484-019-01700-6

2. Kunst AE, Looman CW, Mackenbach JP. The decline in winter excess mortality in The Netherlands. International Journal of Epidemiology 1991;20:971–977. doi:10.1093/ije/20.4.971

3. Ledberg A. A large decrease in the magnitude of seasonal fluctuations in mortality among elderly explains part of the increase in longevity in Sweden during 20th century. BMC Public Health 2020;20:1674. doi:10.1186/s12889-020-09749-4

4. Lerchl A. Changes in the seasonality of mortality in Germany from 1946 to 1995: the role of temperature. Int J Biometeorol 1998;42:84–88. doi:10.1007/s004840050089

For peer review only - http://bmjopen.bmj.com/site/about/guidelines.xhtml

5. Fowler T, Southgate RJ, Waite T, et al. Excess winter deaths in Europe: a multi-country descriptive analysis. Eur J Public Health 2015;25:339–345. doi:10.1093/eurpub/cku073

6. Parks RM, Bennett JE, Foreman KJ, et al. National and regional seasonal dynamics of all-cause and cause-specific mortality in the USA from 1980 to 2016. Elife 2018;7:e35500. doi:10.7554/eLife.35500

7. Davie GS, Baker MG, Hales S, et al. Trends and determinants of excess winter mortality in New Zealand: 1980 to 2000. BMC Public Health 2007;7:263–272. doi:10.1186/1471-2458-7-263

8. Reichert TA, Simonsen L, Sharma A, et al. Influenza and the Winter Increase in Mortality in the United States, 1959–1999. American Journal of Epidemiology 2004;160:492–502. doi:10.1093/aje/kwh227

9. Vestergaard LS, Nielsen J, Krause TG, et al. Excess all-cause and influenza-attributable mortality in Europe, December 2016 to February 2017. Eurosurveillance 2017;22:30506: http://dx.doi.org/10.2807/1560-7917.ES.2017.22.14.30506

10. Gasparrini A, Guo Y, Hashizume M, et al. Mortality risk attributable to high and low ambient temperature: a multicountry observational study. Lancet 2015;386:369–375. doi:10.1016/S0140-6736(14)62114-0

11. Rau R, Bohk-Ewald C, Muszyńska MM, et al. Seasonality of Causes of Death. In: Rau, R, Bohk-Ewald, C, Muszyńska MM, et al. eds. Visualizing Mortality Dynamics in the Lexis Diagram. The Springer Series on Demographic Methods and Population Analysis. Springer Cham 2018:99–122.

12. Rau R. Seasonality in human mortality: a demographic approach. Berlin; New York: Springer 2007.

13. Tanner LM, Moffatt S, Milne EMG, et al. Socioeconomic and behavioural risk factors for adverse winter health and social outcomes in economically developed countries: a systematic review of quantitative observational studies. J Epidemiol Community Health 2013;67:1061–1067. doi:10.1136/jech-2013-202693

14. Almendra R, Santana P, Vasconcelos J. Evidence of social deprivation on the spatial patterns of excess winter mortality. Int J Public Health 2017;62:849–856. doi:10.1007/s00038-017-0964-7

15. Stacey D, Pritchard C. An ecological study of excess winter mortality in England and deprivation. Public Health 2016;141:207–209. doi:10.1016/j.puhe.2016.09.023

16. Phu Pin S, Golmard JL, Cotto E, et al. Excess Winter Mortality in France: Influence of Temperature, Influenza Like Illness, and Residential Care Status. Journal of the American Medical Directors Association 2012;13:309.e1-309.e7. doi:10.1016/j.jamda.2011.06.005

For peer review only - http://bmjopen.bmj.com/site/about/guidelines.xhtml

17. Rolden HJA, Rohling JHT, van Bodegom D, et al. Seasonal Variation in Mortality, Medical Care Expenditure and Institutionalization in Older People: Evidence from a Dutch Cohort of Older Health Insurance Clients. PLoS One 2015;10:e0143154. doi:10.1371/journal.pone.0143154

18. Hajat S, Kovats RS, Lachowycz K. Heat-related and cold-related deaths in England and Wales: who is at risk? Occup Environ Med 2006;64:93–100. doi:10.1136/oem.2006.029017

19. Healy JD. Excess winter mortality in Europe: a cross country analysis identifying key risk factors. Journal of Epidemiology and Community Health 2003;57:784–789. doi:10.1136/jech.57.10.784

20. Gemmell I. Seasonal variation in mortality in Scotland. International Journal of Epidemiology 2000;29:274–279. doi:10.1093/ije/29.2.274

21. Shkolnikov VM, Klimkin I, McKee M, et al. What should be the baseline when calculating excess mortality? New approaches suggest that we have underestimated the impact of the COVID-19 pandemic and previous winter peaks. SSM Popul Health 2022;18:101118. doi:10.1016/j.ssmph.2022.101118

22. Wilkinson P, Pattenden S, Armstrong B, et al. Vulnerability to winter mortality in elderly people in Britain: population based study. BMJ 2004;329:647. doi:10.1136/bmj.38167.589907.55

23. Reinikainen M, Uusaro A, Ruokonen E, et al. Excess mortality in winter in Finnish intensive care. Acta Anaesthesiol Scand 2006;50:706–711. doi:10.1111/j.1399-6576.2006.01041.x

24. OECD. Framework for integrated analysis. In: OECD Framework for Statistics on the Distribution of Household Income, Consumption and Wealth. OECD. 2013;171–192.

25. Muggeo VMR. segmented: An R Package to Fit Regression Models with Broken-Line Relationships. 2008.

26. Donaldson GC, Keatinge WR. Mortality related to cold weather in elderly people in southeast England, 1979-94. BMJ 1997;315:1055–1056. doi:10.1136/bmj.315.7115.1055

27. Ekamper P, Van Poppel F, Van Duin C, et al. 150 Years of temperature-related excess mortality in the Netherlands. Demographic Research 2009;21:385–426. doi:10.4054/DemRes.2009.21.14

28. Näyhä S. Short and Medium-Term Variations in Mortality in Finland: A study on cyclic variations, annual and weekly periods and certain irregular changes in mortality in Finland during the period 1868-1972. Scand J Soc Med Suppl. 1981;21:1-101. PMID:7022620

29. Näyhä S. Seasonal variation of deaths in Finland--is it still diminishing? Int J Circumpolar Health 2000;59: 182–7. PMID:11209665

30. von Klot S, Zanobetti A, Schwartz J. Influenza epidemics, seasonality, and the effects of cold weather on cardiac mortality. Environ Health 2012;11:74. doi:10.1186/1476-069X-11-74

For peer review only - http://bmjopen.bmj.com/site/about/guidelines.xhtml

31. Kinney PL, Schwartz J, Pascal M, et al. Winter season mortality: will climate warming bring benefits? Environ Res Lett 2015;10:064016. doi: 10.1088/1748-9326/10/6/064016

32. Jones RP. Excess winter mortality and stalling international improvements in life expectancy and mortality rates. Brit J Healthc Manage 2020;26:1–8. doi:10.12968/bjhc.2020.0020

33. Voordouw BCG, van der Linden PD, Simonian S, et al. Influenza vaccination in community-dwelling elderly: impact on mortality and influenza-associated morbidity. Arch Intern Med 2003;163:1089–1094. doi:10.1001/archinte.163.9.1089

34. Fireman B, Lee J, Lewis N, et al. Influenza vaccination and mortality: differentiating vaccine effects from bias. Am J Epidemiol 2009;170:650–656. doi:10.1093/aje/kwp173

35. Simonsen L, Taylor RJ, Viboud C, et al. Mortality benefits of influenza vaccination in elderly people: an ongoing controversy. Lancet Infect Dis 2007;7:658–666. doi:10.1016/S1473-3099(07)70236-0

36. Demicheli V, Jefferson T, Pietrantonj CD, et al. Vaccines for preventing influenza in the elderly. Cochrane Database Syst Rev: 2018;2(2):CD004876. doi: 10.1002/14651858.CD004876.pub4

37. Feinstein CA. Seasonality of deaths in the U.S. by age and cause. Demographic Research 2002;6:471–488. doi:10.4054/DemRes.2002.6.17

38. Robine J-M. When climate change encounters the revolution in adult longevity. Aging Clin Exp Res 2017;29:1073–1074. doi:10.1007/s40520-017-0839-z

39. Arbuthnott K, Hajat S, Heaviside C, et al. What is cold-related mortality? A multi-disciplinary perspective to inform climate change impact assessments. Environ Int 2018;121:119–129. doi:10.1016/j.envint.2018.08.053

40. Milte R, Petersen J, Boylan J, et al. Prevalence and determinants of physical frailty among people living in residential aged care facilities: a large-scale retrospective audit. BMC Geriatrics 2022;22:424. doi:10.1186/s12877-022-03101-8

41. Dent E, Ambagtsheer RC, Beilby J, et al. Frailty and Seasonality. J Nutr Health Aging 2020;24:547–549. doi:10.1007/s12603-020-1367-7

42. Liddell C, Morris C, Gray B, et al. Excess winter mortality associated with Alzheimer's Disease and related dementias in the UK: A case for energy justice. Energy Research & Social Science 2016;11:256–262. doi:10.1016/j.erss.2015.11.007

43. Zanobetti A, O'Neill MS, Gronlund CJ, et al. Susceptibility to Mortality in Weather Extremes: Effect Modification by Personal and Small-Area Characteristics. Epidemiology 2013;24:809–819. doi:10.1097/01.ede.0000434432.06765.91

For peer review only - http://bmjopen.bmj.com/site/about/guidelines.xhtml

44. Rocklöv J, Forsberg B, Ebi K, et al. Susceptibility to mortality related to temperature and heat and cold wave duration in the population of Stockholm County, Sweden. Global Health Action 2014;7:22737. doi:10.3402/gha.v7.22737

45. Strongman H, Carreira H, De Stavola BL, et al. Factors associated with excess all-cause mortality in the first wave of the COVID-19 pandemic in the UK: A time series analysis using the Clinical Practice Research Datalink. PLoS Med 2022;19:e1003870. doi: 10.1371/journal.pmed.1003870

46. Schwartz J. Who is Sensitive to Extremes of Temperature? A Case-Only Analysis. Epidemiology 2005;16:67–72. doi:10.1097/01.ede.0000147114.25957.71

47. Tammes P, Sartini C, Preston I, et al. Use of primary care data to predict those most vulnerable to cold weather: a case-crossover analysis. Br J Gen Pract 2018;68:e146–e156. doi:10.3399/bjgp18X694829

48. Bhaskaran K, Hajat S, Haines A. et al. Short term effects of temperature on risk of myocardial infarction in England and Wales: time series regression analysis of the Myocardial Ischaemia National Audit Project (MINAP) registry. BMJ 2010;341:c3823. doi:10.1136/bmj.c3823

49. van Rossum CT, Shipley MJ, Hemingway H, et al. Seasonal variation in cause-specific mortality: Are there high-risk groups? 25-year follow-up of civil servants from the first Whitehall study. International Journal of Epidemiology 2001;30:1109–1116. doi:10.1093/ije/30.5.1109

50. Barnett AG, Dobson AJ, McElduff P. Cold periods and coronary events: an analysis of populations worldwide. J Epidemiol Community Health 2005;59:551–557. doi:10.1136/jech.2004.028514

51. Gerber Y, Jacobsen SJ, Killian JM. Seasonality and Daily Weather Conditions in Relation to Myocardial Infarction and Sudden Cardiac Death in Olmsted County, Minnesota, 1979 to 2002. J Am Coll Cardiol 2006;48:287–292. doi:10.1016/j.jacc.2006.02.065

52. Mackenbach JP. Health inequalities: Persistence and change in modern welfare states. 1st ed. Oxford University Press. 2019.

53. Hales S, Blakely T, Foster RH, et al. Seasonal patterns of mortality in relation to social factors. J Epidemiol Community Health 2012;66:379–384. doi:10.1136/jech.2010.111864

54. Turunen M, Iso-Markku K, Pekkonen M, et al. Statistical associations between housing quality and health among Finnish households with children - Results from two (repeated) national surveys. Sci Total Environ 2017;574:1580–1587. doi:10.1016/j.scitotenv.2016.08.194

55. Thompson WW, Shay DK, Weintraub E, et al. Mortality Associated With Influenza and Respiratory Syncytial Virus in the United States. JAMA 2003;289:179–186. doi:10.1001/jama.289.2.179

For peer review only

For peer review only - http://bmjopen.bmj.com/site/about/guidelines.xhtml

56. Warren-Gash C, Blackburn R, Whitaker H, et al. Laboratory-confirmed respiratory infections as triggers for acute myocardial infarction and stroke: a self-controlled case series analysis of national linked datasets from Scotland. Eur Respir J 2018;51:1701794. doi:10.1183/13993003.01794-2017

Figure 1. All-cause winter and nonwinter mortality rates, Finland 1971-2019, men and women aged 60 and over.

Figure 2. Rate differences (upper panel) and rate ratios (lower panel) of winter and nonwinter mortality rates with detected breakpoints (vertical dashed lines) and their confidence intervals, Finland 1971-2019, men and women aged 60 and over.

For peer review only

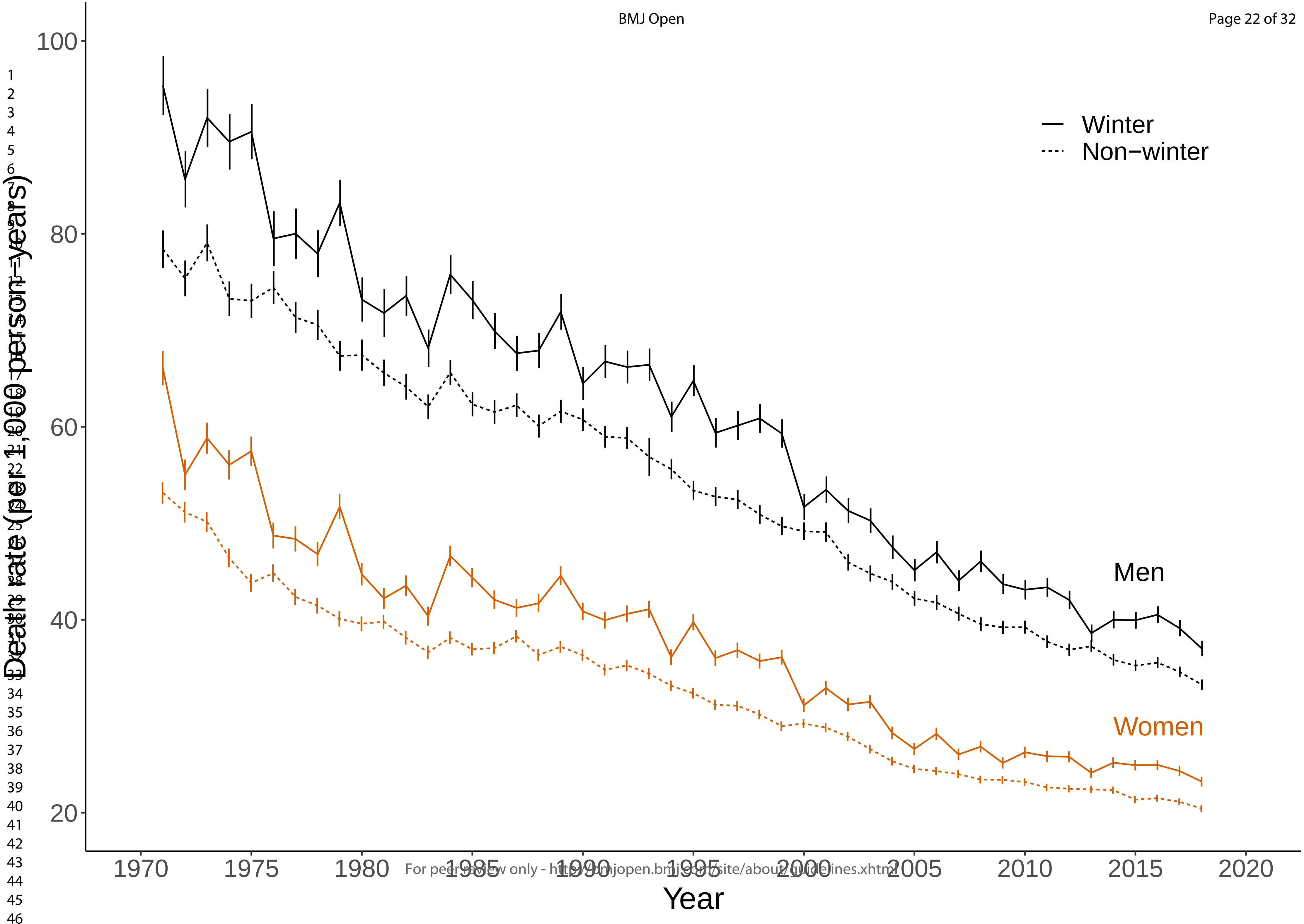
Winter
Non−winter
Men
Women
Death rate (per 1,000 person−years)
100
80
60
40
20
1970
1975
1980
1985
1990
1995
2000
2005
2010
2015
2020
Year

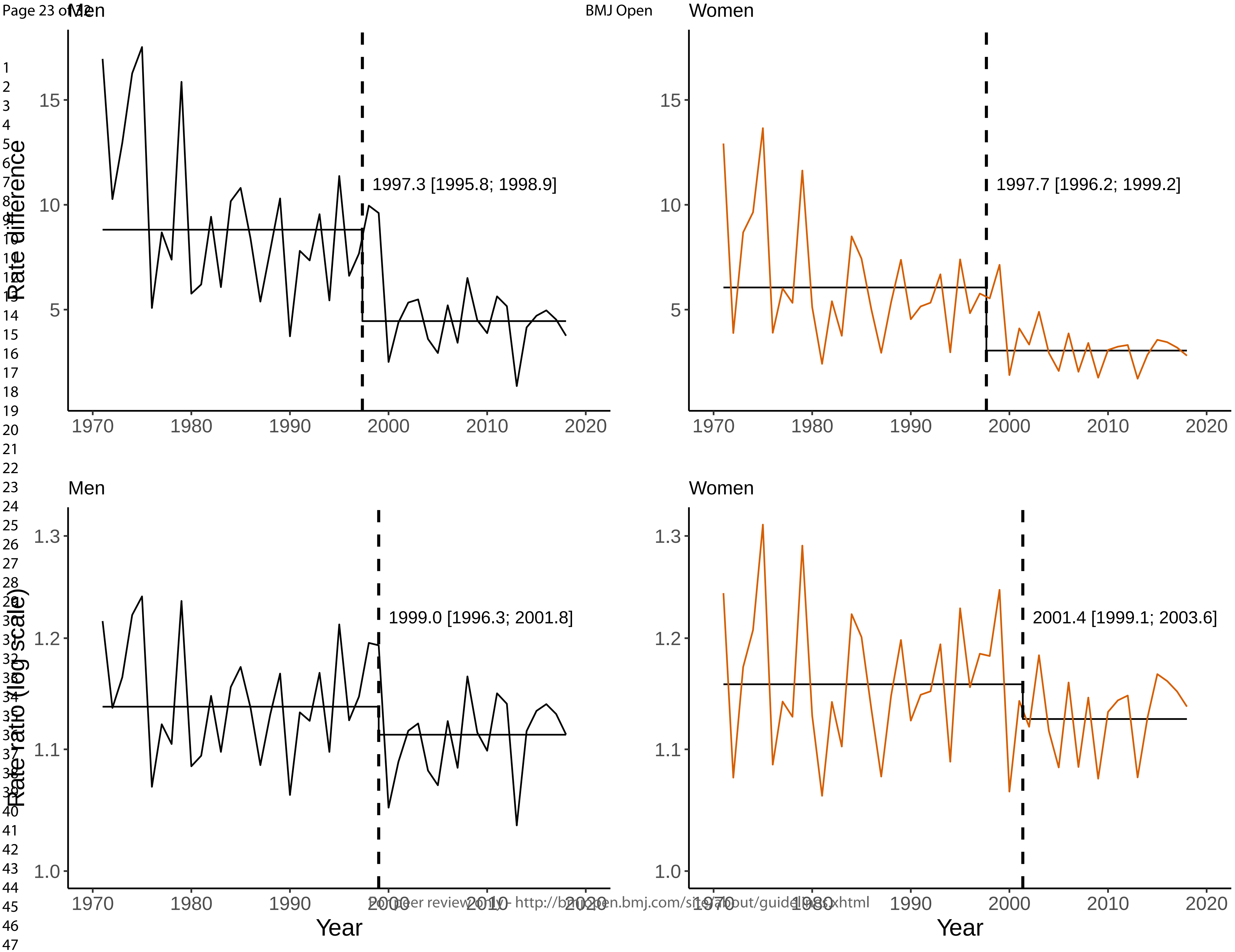
Men
Women
Rate difference
1997.3 [1995.8; 1998.9]
1997.7 [1996.2; 1999.2]
15
10
5
1970
1980
1990
2000
2010
2020
Men
Women
Rate ratio (log scale)
1999.0 [1996.3; 2001.8]
2001.4 [1999.1; 2003.6]
1.3
1.2
1.1
1.0
Year
Year

SUPPLEMENTAL MATERIAL

Excess winter mortality in Finland in 1971-2019 – a register-based study on long-term trends and effect modification by sociodemographic characteristics and pre-existing health conditions

Ulla Suulamo, Hanna Remes, Lasse Tarkiainen, Mike Murphy, and Pekka Martikainen

Table S1. Categories of pre-existing health conditions.

Condition	Health care diagnosis, ICD-10 (Care register for health care HILMO, THL)	Refund categories, Finnish disease, or medicine codes (Register of Reimbursements for medicine purchases, Kela)
Dementia	F00-F03, F05.1, G30	307
Mental health disorders (excl. alcohol related)	F04-F99, excl. F10	Severe psychosis and other severe mental disorders (112)<br>Behavioural disorders in people with intellectual disabilities (113)
Diseases of the nervous system	G00-G29, G31-G99, excl. G31.2, G40.51, G62.1, G72.1	Parkinson's disease (110)<br>Epilepsy (111)<br>Multiple sclerosis (109)<br>Myasthenia gravis (108)<br>Neuralgia trigemini, neuralgia glossopharyngeal (119)<br>Narcolepsy (214)<br>Dimethyl fumarate, diroximel fumarate etc (157)<br>Retigabine and vigabatrin (181)<br>Brivaracetam, eslikarbazepin etc (182)<br>Lamotrigin and topiramate (183)<br>Rasagiline (193)<br>Rufinamide (199)
Diabetes	E10-E14	Diabetes (103)<br>Diabetes other than insulin therapy (215)
Cancer	C00-C97	Prostate cancer (116)<br>Breast cancer (115)
Diseases of the circulatory system (excl. alcohol related)	I00-I99, excl. I42.6	Chronic cardiac insufficiency (201)<br>Chronic coronary heart disease (206)<br>Chronic hypertension (205)<br>Chronic cardiac arrhythmias (207)
Diseases of the respiratory system	J00-J99, excl. J65	Chronic asthma and COPD (203)
Alcohol related causes	F10, G31.2, G40.51, G62.1, G72.1, I42.6, K29.2, K70, K86.0, K85.2, O35.4, X45	
Accidents and violence (excl. alcohol related)	V01-Y89, excl. X45	

For peer review only - http://bmjopen.bmj.com/site/about/guidelines.xhtml

Table S2. Changes in sociodemographic characteristics and pre-existing health conditions between 2000 and 2018, by age category, population aged 60 and over.

	2000/01 (%)				2018/19 (%)			
	**60–69**	**70–79**	**80–89**	**90–**	**60–69**	**70–79**	**80–89**	**90–**
**Sociodemographic characteristics**								
Mean age (sd)	64.3 (2.9)	74.1 (2.8)	83.6 (2.8)	92.2 (2.3)	64.5 (2.9)	73.8 (2.9)	83.8 (2.8)	92.5 (2.5)
Sex								
Men	47.0	39.0	28.1	20.9	48.5	45.6	37.6	24.9
Women	53.0	61.0	71.9	79.1	51.5	54.4	62.4	75.1
Income quartile								
1 (lowest)	25.0	25.0	25.0	25.2	25.0	25.0	25.0	24.8
2	25.0	25.0	25.0	24.9	25.0	25.0	25.0	25.0
3	25.0	25.0	25.0	24.9	25.0	25.0	25.0	25.1
4 (highest)	25.0	25.0	25.0	24.9	25.0	25.0	25.0	25.0
Living arrangement								
Household of >1	74.6	60.7	39.3	25.5	70.2	65.3	46.4	23.5
Alone	24.5	37.0	51.2	47.0	28.2	32.8	47.2	57.5
Institution	1.0	2.3	9.5	27.4	1.5	1.9	6.4	19.1
Large regions								
Coast	37.1	35.0	36.8	40.8	38.9	39.6	36.6	36.8
South	28.0	29.7	30.4	29.7	27.0	28.1	28.9	30.6
Center	19.0	19.6	18.9	17.7	17.8	17.6	18.9	18.2
North	16.0	15.7	13.9	11.8	16.3	14.7	15.7	14.4
**Pre-existing health conditions**								
Dementia	0.2	1.5	4.4	7.2	0.4	2.5	12.0	22.4
Mental health disorders	3.7	3.8	3.9	2.9	4.0	3.6	3.8	3.5
Cancer	3.3	5.1	5.7	4.4	5.1	8.8	11.0	9.3
Diseases of the nervous system	3.9	4.9	5.2	4.1	6.3	7.2	7.3	4.9
Diabetes	5.8	8.7	9.7	7.7	12.4	17.4	18.1	13.7
Circulatory	35.3	49.7	58.6	57.8	24.2	39.9	54.5	58.6
Respiratory	8.2	10.4	10.9	10.3	9.4	12.0	14.9	15.7
Alcohol related causes	0.4	0.2	0.1	0.1	0.7	0.4	0.2	0.0
Accidents and violence	3.3	4.4	7.2	10.2	5.0	6.2	9.6	13.7
Other than specified above	23.0	30.0	36.7	34.7	27.1	36.1	44.2	43.2
Any health condition	57.5	70.8	79.1	78.5	57.0	71.7	83.9	87.0
Total	100.0	100.0	100.0	100.0	100.0	100.0	100.0	100.0
N	488,751	372,305	152,561	22,026	729,831	530,492	248,299	50,543

For peer review only

For peer review only - http://bmjopen.bmj.com/site/about/guidelines.xhtml

Table S3. Relative risks of excess winter mortality by individual characteristics (95% CI) by age category. Finland 2000-2019, men and women aged 60 and over.

	**60–69** n=135,380	**70–79** n=224,573	**80–89** n=325,160	**90 and over** n=139,811
Winter-nonwinter rate ratio	1.06 (1.05, 1.07)	1.10 (1.09, 1.11)	1.14 (1.13, 1.15)	1.19 (1.17, 1.20)
**Sociodemographic characteristics**				
Sex				
Men	1.00	1.00	1.00	1.00
Women	1.01 (0.99, 1.03)	1.01 (0.99, 1.03)	**1.02 (1.00, 1.03)**	0.99 (0.97, 1.02)
Income quartile				
1 (lowest)	1.00	1.00	1.00	1.00
2	0.99 (0.96, 1.02)	1.00 (0.98, 1.03)	1.00 (0.98, 1.02)	1.01 (0.98, 1.04)
3	**0.96 (0.93, 0.99)**	1.01 (0.98, 1.03)	1.00 (0.98, 1.02)	1.00 (0.97, 1.03)
4 (highest)	0.98 (0.94, 1.01)	0.99 (0.96, 1.01)	1.00 (0.98, 1.02)	1.00 (0.97, 1.03)
Living arrangement				
Household of >1	1.00	1.00	1.00	1.00
Alone	1.01 (0.99, 1.03)	1.00 (0.98, 1.01)	1.01 (0.99, 1.02)	0.98 (0.96, 1.01)
Institution	**1.09 (1.04, 1.15)**	**1.07 (1.04, 1.10)**	**1.03 (1.01, 1.05)**	1.01 (0.98, 1.04)
Region				
Coast	1.00	1.00	1.00	1.00
South	1.02 (0.99, 1.05)	1.02 (1.00, 1.04)	1.01 (0.99, 1.03)	1.01 (0.98, 1.04)
Center	1.00 (0.97, 1.04)	1.02 (1.00, 1.05)	1.00 (0.98, 1.02)	1.00 (0.97, 1.03)
North	1.03 (0.99, 1.06)	1.02 (1.00, 1.05)	1.02 (1.00, 1.04)	1.01 (0.97, 1.04)
**Pre-existing health conditions**				
Dementia				
No	1.00	1.00	1.00	1.00
Yes	**1.07 (1.00, 1.15)**	**1.07 (1.04, 1.10)**	**1.04 (1.02, 1.05)**	1.01 (0.98, 1.03)
Mental health disorders				
No	1.00	1.00	1.00	1.00
Yes	**1.06 (1.02, 1.10)**	**1.04 (1.01, 1.07)**	1.02 (0.99, 1.05)	0.98 (0.93, 1.04)
Cancer				
No	1.00	1.00	1.00	1.00
Yes	**0.94 (0.91, 0.97)**	**0.94 (0.92, 0.96)**	**0.93 (0.91, 0.95)**	**0.94 (0.90, 0.98)**
Diseases of the nervous system				
No	1.00	1.00	1.00	1.00
Yes	**1.05 (1.01, 1.08)**	0.99 (0.97, 1.02)	0.99 (0.97, 1.02)	0.99 (0.94, 1.04)
Diabetes				
No	1.00	1.00	1.00	1.00
Yes	1.00 (0.97, 1.03)	1.02 (0.99, 1.04)	**1.02 (1.00, 1.04)**	1.00 (0.97, 1.04)
Circulatory				
No	1.00	1.00	1.00	1.00
Yes	0.99 (0.96, 1.01)	1.00 (0.99, 1.02)	1.00 (0.99, 1.02)	0.99 (0.97, 1.02)
Respiratory				
No	1.00	1.00	1.00	1.00
Yes	**1.03 (1.00, 1.06)**	**1.03 (1.01, 1.05)**	**1.02 (1.00, 1.04)**	1.01 (0.98, 1.04)
Alcohol related causes				
No	1.00	1.00	1.00	1.00
Yes	0.98 (0.92, 1.04)	0.98 (0.90, 1.07)	0.87 (0.73, 1.05)	1.19 (0.68, 2.08)
Accidents and violence				
No	1.00	1.00	1.00	1.00
Yes	0.99 (0.95, 1.03)	0.99 (0.96, 1.02)	1.00 (0.98, 1.02)	1.02 (0.98, 1.05)
Other than specified above				
No	1.00	1.00	1.00	1.00
Yes	1.00 (0.98, 1.02)	**0.96 (0.94, 0.98)**	0.99 (0.97, 1.00)	**0.97 (0.95, 0.99)**
Any health condition				
No	1.00	1.00	1.00	1.00
Yes	0.99 (0.96, 1.02)	1.00 (0.97, 1.02)	1.01 (0.99, 1.04)	0.97 (0.94, 1.00)

Note: Relative risks are the interaction terms of the winter indicator and the effect modifier. These show the relative differences in the effect of winter on mortality between effect modifier categories. Models are adjusted for sex, age, income, living arrangement, region, and year.

For peer review only - http://bmjopen.bmj.com/site/about/guidelines.xhtml

Table S4. Relative risks of excess winter mortality by selected individual characteristics, whether living in institutions or community. Finland 2000-2019, men and women aged 60 and over.

	Winter-nonwinter interaction (95% CI)	
	**In institution** n=156,851	**In community** n=668,073
Winter-nonwinter rate ratio	1.18 (1.16, 1.19)	1.11 (1.10, 1.12)
**Sociodemographic characteristics**		
Sex		
Men	1.00	1.00
Women	1.01 (0.99, 1.03)	**1.02 (1.01, 1.04)**
Age category		
60–69	1.00	1.00
70–79	1.02 (0.97, 1.07)	**1.03 (1.02, 1.05)**
80–89	1.01 (0.96, 1.06)	**1.07 (1.06, 1.09)**
90 and over	1.05 (1.00, 1.10)	**1.11 (1.09, 1.13)**
**Pre-existing health conditions**		
Dementia		
No	1.00	1.00
Yes	1.00 (0.98, 1.02)	**1.06 (1.05, 1.08)**
Respiratory		
No	1.00	1.00
Yes	1.01 (0.98, 1.04)	**1.03 (1.01, 1.04)**

Note: Relative risks are the interaction terms of the winter indicator and the effect modifier. These show the relative differences in the effect of winter on mortality between effect modifier categories. Models are adjusted for sex, age, income, living arrangement, region, and year.

For peer review only

The RECORD statement – checklist of items, extended from the STROBE statement, that should be reported in observational studies using routinely collected health data.

	Item No.	STROBE items	Location in manuscript where items are reported	RECORD items	Location in manuscript where items are reported
**Title and abstract**					
	1	(a) Indicate the study's design with a commonly used term in the title or the abstract (b) Provide in the abstract an informative and balanced summary of what was done and what was found	page 1-2	RECORD 1.1: The type of data used should be specified in the title or abstract. When possible, the name of the databases used should be included.<br><br>RECORD 1.2: If applicable, the geographic region and timeframe within which the study took place should be reported in the title or abstract.<br><br>RECORD 1.3: If linkage between databases was conducted for the study, this should be clearly stated in the title or abstract.	page 1-2
**Introduction**					
Background rationale	2	Explain the scientific background and rationale for the investigation being reported	page 3		
Objectives	3	State specific objectives, including any prespecified hypotheses	page 4		
**Methods**					
Study Design	4	Present key elements of study design early in the paper	page 4		
Setting	5	Describe the setting, locations, and relevant dates, including periods of recruitment, exposure, follow-up, and data collection	page 4		

For peer review only

Participants	6	*(a) Cohort study* - Give the eligibility criteria, and the sources and methods of selection of participants. Describe methods of follow-up<br>*Case-control study* - Give the eligibility criteria, and the sources and methods of case ascertainment and control selection. Give the rationale for the choice of cases and controls<br>*Cross-sectional study* - Give the eligibility criteria, and the sources and methods of selection of participants<br><br>*(b) Cohort study* - For matched studies, give matching criteria and number of exposed and unexposed<br>*Case-control study* - For matched studies, give matching criteria and the number of controls per case	page 4	RECORD 6.1: The methods of study population selection (such as codes or algorithms used to identify subjects) should be listed in detail. If this is not possible, an explanation should be provided.<br><br>RECORD 6.2: Any validation studies of the codes or algorithms used to select the population should be referenced. If validation was conducted for this study and not published elsewhere, detailed methods and results should be provided.<br><br>RECORD 6.3: If the study involved linkage of databases, consider use of a flow diagram or other graphical display to demonstrate the data linkage process, including the number of individuals with linked data at each stage.	page 4
Variables	7	Clearly define all outcomes, exposures, predictors, potential confounders, and effect modifiers. Give diagnostic criteria, if applicable.	page 4-5	RECORD 7.1: A complete list of codes and algorithms used to classify exposures, outcomes, confounders, and effect modifiers should be provided. If these cannot be reported, an explanation should be provided.	
Data sources/ measurement	8	For each variable of interest, give sources of data and details of methods of assessment (measurement).<br>Describe comparability of assessment methods if there is more than one group	page 4-5		

For peer review only

Bias	9	Describe any efforts to address potential sources of bias	page 13		
Study size	10	Explain how the study size was arrived at			
Quantitative variables	11	Explain how quantitative variables were handled in the analyses. If applicable, describe which groupings were chosen, and why	page 4-5		
Statistical methods	12	(a) Describe all statistical methods, including those used to control for confounding<br>(b) Describe any methods used to examine subgroups and interactions<br>(c) Explain how missing data were addressed<br>(d) *Cohort study* - If applicable, explain how loss to follow-up was addressed<br>*Case-control study* - If applicable, explain how matching of cases and controls was addressed<br>*Cross-sectional study* - If applicable, describe analytical methods taking account of sampling strategy<br>(e) Describe any sensitivity analyses	page 5-6		
Data access and cleaning methods		..		RECORD 12.1: Authors should describe the extent to which the investigators had access to the database population used to create the study population.	

For peer review only

				RECORD 12.2: Authors should provide information on the data cleaning methods used in the study.	
Linkage		..		RECORD 12.3: State whether the study included person-level, institutional-level, or other data linkage across two or more databases. The methods of linkage and methods of linkage quality evaluation should be provided.	page 5
**Results**					
Participants	13	(a) Report the numbers of individuals at each stage of the study (*e.g.*, numbers potentially eligible, examined for eligibility, confirmed eligible, included in the study, completing follow-up, and analysed)<br>(b) Give reasons for non-participation at each stage.<br>(c) Consider use of a flow diagram	page 7	RECORD 13.1: Describe in detail the selection of the persons included in the study (*i.e.,* study population selection) including filtering based on data quality, data availability and linkage. The selection of included persons can be described in the text and/or by means of the study flow diagram.	page 4-5
Descriptive data	14	(a) Give characteristics of study participants (*e.g.*, demographic, clinical, social) and information on exposures and potential confounders<br>(b) Indicate the number of participants with missing data for each variable of interest<br>(c) *Cohort study* - summarise follow-up time (*e.g.*, average and total amount)	page 7		
Outcome data	15	*Cohort study* - Report numbers of outcome events or summary measures over time<br>*Case-control study* - Report numbers in each exposure	page 9		

For peer review only

		category, or summary measures of exposure<br>*Cross-sectional study* - Report numbers of outcome events or summary measures			
Main results	16	(a) Give unadjusted estimates and, if applicable, confounder-adjusted estimates and their precision (e.g., 95% confidence interval). Make clear which confounders were adjusted for and why they were included<br>(b) Report category boundaries when continuous variables were categorized<br>(c) If relevant, consider translating estimates of relative risk into absolute risk for a meaningful time period	page 9		
Other analyses	17	Report other analyses done—e.g., analyses of subgroups and interactions, and sensitivity analyses	page 8		
**Discussion**					
Key results	18	Summarise key results with reference to study objectives	page 10		
Limitations	19	Discuss limitations of the study, taking into account sources of potential bias or imprecision. Discuss both direction and magnitude of any potential bias	page 13	RECORD 19.1: Discuss the implications of using data that were not created or collected to answer the specific research question(s). Include discussion of misclassification bias, unmeasured confounding, missing data, and changing eligibility over time, as they pertain to the study being reported.	page 13
Interpretation	20	Give a cautious overall interpretation of results considering objectives,	page 13		

For peer review only

		limitations, multiplicity of analyses, results from similar studies, and other relevant evidence			
Generalisability	21	Discuss the generalisability (external validity) of the study results	page 13		
**Other Information**					
Funding	22	Give the source of funding and the role of the funders for the present study and, if applicable, for the original study on which the present article is based	page 13		
Accessibility of protocol, raw data, and programming code		..		RECORD 22.1: Authors should provide information on how to access any supplemental information such as the study protocol, raw data, or programming code.	page 14

*Reference: Benchimol EI, Smeeth L, Guttmann A, Harron K, Moher D, Petersen I, Sørensen HT, von Elm E, Langan SM, the RECORD Working Committee. The REporting of studies Conducted using Observational Routinely-collected health Data (RECORD) Statement. *PLoS Medicine* 2015; in press.

*Checklist is protected under Creative Commons Attribution (CC BY) license.

For peer review only
